# Study protocol for a multiarm, randomized controlled trial to determine the effectiveness of community-based frailty rehabilitation to improve physical function in older adults: The OPTIMAL Fitness Trial

**DOI:** 10.1371/journal.pone.0343338

**Published:** 2026-03-12

**Authors:** Alexandra Papaioannou, Courtney Kennedy, Justin Lee, Patricia Hewston, Caitlin McArthur, Shyam Maharaj, Jonathan Adachi, Pauline Boulos, Raja Bobba, Alexander Rabinovich, Brian McKenna, Lisa Palubiski, Dee Mangin, Lehana Thabane, Sharon Marr, Sharon Kaasalainen, Jean-Éric Tarride, Olga Theou, David Armstrong, Ahmed Negm, Michael Noseworthy, Kenneth Rockwood, Lisa Dolovich, Hajar Abu Alrob, Genevieve Hladysh, Karen Thompson, George Ioannidis

**Affiliations:** 1 Department of Medicine, McMaster University, Hamilton, Ontario, Canada; 2 Department of Health Research Methods, Evidence and Impact (HEI), McMaster University, Hamilton, Ontario, Canada; 3 Geras Centre for Aging Research, Hamilton Health Sciences, St. Peter’s Hospital, Hamilton, Ontario, Canada; 4 School of Rehabilitation Science, McMaster University, Hamilton, Ontario, Canada; 5 School of Physiotherapy, Dalhousie University, Halifax, Nova Scotia, Canada; 6 Hamilton Health Sciences, Hamilton, Ontario, Canada; 7 Department of Surgery, McMaster University, Hamilton, Ontario, Canada; 8 Escarpment Health Centre, Hamilton, Ontario, Canada; 9 Department of Family Medicine, University of Otago, Christchurch, New Zealand; 10 Biostatistics Unit, St Joseph’s Healthcare Hamilton, Hamilton, Ontario, Canada; 11 Faculty of Health Sciences, University of Johannesburg, Johannesburg, South Africa; 12 School of Nursing, McMaster University, Hamilton, Ontario, Canada; 13 Centre for Health Economics and Policy Analysis, McMaster University, Hamilton, Ontario, Canada; 14 Programs for Assessment of Technology in Health, The Research Institute of St. Joe’s Hamilton, St. Joseph’s Healthcare Hamilton, Hamilton, Canada; 15 Department of Medicine, Dalhousie University, Halifax, Nova Scotia, Canada; 16 Cumming School of Medicine, University of Calgary, Calgary, Alberta, Canada; 17 School of Biomedical Engineering, McMaster University, Hamilton, Ontario, Canada; 18 Department of Electrical and Computer Engineering, McMaster University, Hamilton, Ontario, Canada; 19 Leslie Dan Faculty of Pharmacy, University of Toronto, Toronto, Ontario, Canada; 20 YMCA of Hamilton/Burlington/Brantford, Hamilton, Ontario, Canada; Tekirdag Namik Kemal University: Tekirdag Namik Kemal Universitesi, TÜRKIYE

## Abstract

**Background:**

Frailty in older adults is on the rise with the rapid aging population. Frailty is dynamic and proven to be reversible or treatable. Evidence regarding the clinical effectiveness of community-based frailty interventions is limited. Frailty rehabilitation has the potential to be an accessible community-based intervention that may enable independence. We will examine a model of frailty rehabilitation and consider key components for improving physical function, frailty, sarcopenia, and cost-effectiveness.

**Methods:**

A multiarm, parallel-group, partially blinded, randomized controlled trial will compare the clinical and cost-effectiveness of OPTimizing Independence, Mobility, and Active Life (OPTIMAL) Fitness multimodal (group exercise, nutritional coaching/supplementation, medication optimization) versus OPTIMAL Fitness exercise-only and control. Participants in the OPTIMAL Fitness multimodal and exercise-only groups will attend exercise classes twice weekly for 4 months and be provided with a tailored one-hour home exercise program. We will recruit community-dwelling older adults living with frailty aged ≥65 years. Eligibility includes a FRAIL scale score of ≥2 (prefrail or frail), ability to ambulate 25 meters, follow two-step commands, and not attend another group exercise program. The target sample size is 324 participants (n = 108 per arm). The primary outcome is physical function measured via the Short Performance Physical Battery and the 400m Walk Test. Secondary outcomes include frailty change, sarcopenia, cognition, affect, quality of life, and healthcare utilization. Outcome analysis will be performed via generalized linear model analysis. Analyses will be based on the intention-to-treat principle. This protocol is guided by the Standard Protocol Items: Recommendations for Interventional Trials statement.

**Discussion:**

This trial is the first to determine whether a community-based frailty rehabilitation intervention can improve physical function in older adults with frailty and the level of intervention needed. The results from the OPTIMAL Fitness study will guide the development of a standardized program that could be implemented in global regions.

**Trial registration:**

ClinicalTrials.gov Identifier: NCT03824106 Registered January 31, 2019, https://www.clinicaltrials.gov/study/NCT03824106

## Introduction

### Background and rationale {6a}

Frailty is an age-related multidimensional state with multiple causes and contributors and is characterized by diminished strength, endurance, and reduced physiologic function [1–3]. As deficits (symptoms, signs, illnesses, and disabilities) accumulate, older adults become more susceptible to adverse health outcomes, including delirium, falls, disability, hospitalization, and death [1,2,4,5]. These events are often triggered by relatively minor stressor events (e.g., changes in medication, urinary tract infection), which lead to sudden changes in health [5,6]. Our research suggests that approximately 23% of Canadians over the age of 65 are frail [7], and by the age of 85, this estimate increases to over 40% [8]. Older adults who are frail are high users of healthcare services [9], and those with moderate or severe frailty have an 8-fold greater relative risk of institutionalization (95% CI = 4.9--15.2) [10]. Frailty is dynamic in nature (i.e., may improve or worsen over time) [8] and has been demonstrated to be reversible or treatable [11].

Frailty and sarcopenia are distinct but interrelated disease processes with similar causalities, consequences, and treatment indications [12]. Sarcopenia is a progressive and generalized skeletal muscle disorder that is characterized by loss of muscle mass and strength and poor physical performance (i.e., whole-body function related to locomotion) [13]. Sarcopenia is a contributor to the development of physical frailty [13]; conversely, frailty may accelerate the development of sarcopenia [5]. Sarcopenia may also be secondary to other causal factors, including physical inactivity, disease-related immobility, and/or inadequate intake of energy or protein [13].

Frailty and sarcopenia treatment recommendations include exercise, oral nutritional supplementation, and vitamin D3 [14]. The reduction in polypharmacy is an additional important component of frailty management as part of a comprehensive geriatric assessment (CGA). Our network meta-analyses [15,16] demonstrated that physical activity interventions with or without nutritional supplementation are most effective; however, the quality of evidence is low, and more robust randomized controlled trials (RCTs) are needed. Frailty intervention trials have demonstrated that multimodal interventions based on geriatric and rehabilitation services significantly reduce frailty; however, these interventions require specialized assessment teams and are resource intensive [17]. The Frailty Intervention Trials utilized clinical teams for interventions, including clinical nutritional teams, physiotherapist teams, psychiatric teams, and geriatric and rehabilitation specialists, to conduct the trial [17]. Furthermore, it could not be determined whether exercise and nutrition alone are sufficient or whether additional CGA interventions, including polypharmacy management, are needed for adults with frailty. With our rapidly aging population and limited access to specialized geriatric services, developing accessible, cost-effective, community-based approaches to managing frailty is imperative for health systems. Our pilot work has demonstrated that delivering a community-based frailty intervention is feasible in Canada without the additional need for specialized assessment teams [18–20].

Although exercise is widely accepted as a beneficial means to improve physical function, frailty, and sarcopenia, there is limited evidence regarding the benefits of exercise alone in older adults with frailty. Therefore, this study will address whether a tailored group exercise intervention is enough to improve outcomes or whether additional components of frailty management (i.e., nutritional supplementation, medication optimization) are needed.

Our frailty rehabilitation model is based on international consensus guidelines that recommend a multimodal approach to reduce frailty [14] and the World Health Organization Public Health Framework for Healthy Ageing [21] to align community-based health services to meet the complex needs of older adults who are frail. This framework for action is built around the concept of building intrinsic capacity (defined as all the physical and mental capacities that an individual can draw on at any point in time). Intrinsic capacity can be enhanced by promoting behaviours within a supportive environment to enable functional ability (defined as health-related attributes that enable people to be and to do what they have reason to value) [21]. Drawing on the principles of a standardized approach to cardiac [22,23] and cancer rehabilitation [24,25], we have developed a structured frailty rehabilitation program called OPTIMAL Fitness (from the acronym: OPTimizing Independence, Mobility and Active Life). The Medical Research Council guidance on complex interventions supported the evaluation of the OPTIMAL Fitness program [26].

### Objectives {7}

The purpose of this study is to determine whether OPTIMAL Fitness exercise or OPTIMAL Fitness multimodal interventions improve physical function over four months (as measured by the Short Physical Performance Battery (SPPB) and 400m Walk Test) versus control in community-dwelling older adults who are prefrail or frail. The secondary outcomes are to examine the impact of the interventions on frailty, sarcopenia, cognition, mood, activities of daily living, health-related quality of life (HR-QOL), and healthcare utilization (emergency room visits; hospitalization; home care service use; institutionalization). A subset of participants with sarcopenia will undergo (dual-energy X-ray absorptiometry) DXA and magnetic resonance imaging (MRI) scans to determine how the OPTIMAL Fitness exercise or OPTIMAL fitness multimodal interventions affect skeletal muscle biomarkers and body composition. We hypothesize that both the OPTIMAL Fitness exercise and OPTIMAL Fitness multimodal interventions will improve physical function, frailty, and sarcopenia in older adults with frailty and that the multimodal arm will offer greater benefits than the other arms. For the sarcopenia substudy, we hypothesize that both interventions will improve skeletal muscle biomarkers and muscle mass in older adults with sarcopenia, with the multimodal arm again offering enhanced benefits relative to the other arms. In addition, we aim to better understand the physical and functional capabilities of older adults with frailty, including their experiences with the program, and to make recommendations regarding the key elements of the OPTIMAL Fitness frailty rehabilitation model.

### Trial design {8}

This study protocol describes an investigator-initiated, multiarm, parallel-group, partially blinded RCT in Southern Ontario, Canada, conducted by the Geras Centre for Aging Research, which is affiliated with Hamilton Health Sciences and McMaster University. An innovative feature of this intervention lies in the partnership between Hamilton Health Sciences, McMaster University, and the YMCA, which crosses sectors to create sustainable evidence-based community programs. Community-dwelling older adults (aged ≥65 years) with prefrailty or frailty will be randomized into one of three arms: control (usual care plus vitamin D) (Arm 1), OPTIMAL Fitness exercise only (Arm 2), or OPTIMAL Fitness multimodal (Arm 3) and stratified by sex and age for each cohort. Primary and secondary outcomes will be assessed in person by blinded assessors at baseline and four months, with healthcare utilization outcomes collected for an additional six months via telephone follow-up. This protocol is reported in accordance with the Standard Protocol Items: Recommendations for Intervention Trials guidelines [27].

### Participant timeline {13}

**Fig 1:**
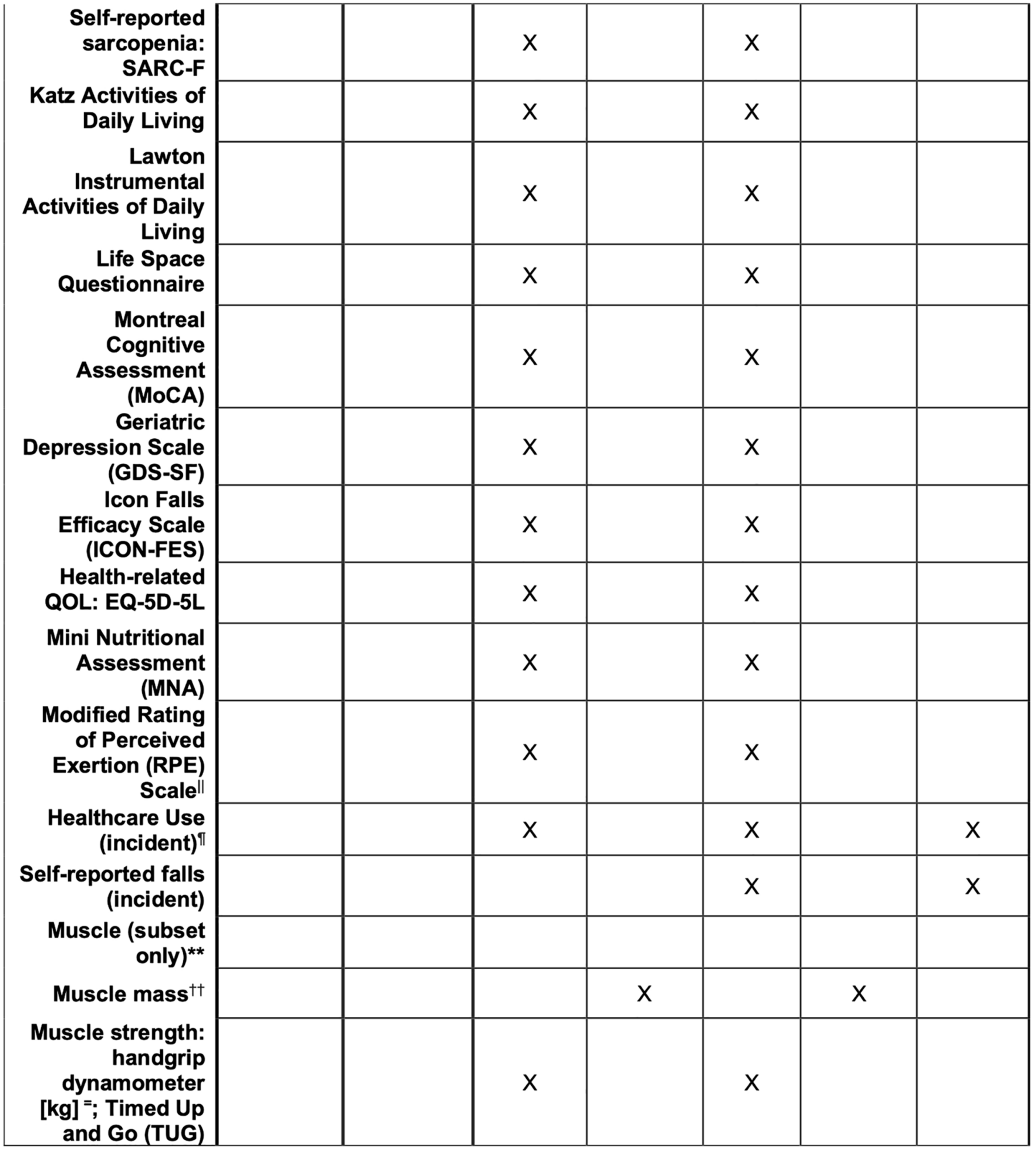
Schedule of enrolment, interventions, and assessments. *Age, sex, gender, housing, education, smoking status, chronic conditions, past surgeries, falls and fracture history, healthcare utilization past 3 months, mobility aids, and participation in other activation/social programs. † Height and weight, heart rate, and blood pressure. ‡ Medications, dietary/oral nutritional supplements, vitamins/minerals. § Exercise barriers, side effects to exercise, barriers to transportation to the YMCA, facilitators/interests in having a healthy level of activity and eating well. || Fitness measure; post-400m walk test. ¶ Number of visits to primary care provider, walk-in clinic, specialist visits, physiotherapist, urgent care, emergency department; hospitalizations. Collect past 3 months at the baseline assessment. ** Up to 75 participants screened for sarcopenia based on the AWGS guidelines undergo dual-energy X-ray absorptiometry (DXA), and a subset of 36 participants of those 75 participants will undergo an MRI at a time (max. 1 month) separate from the primary baseline assessments. Participants who are invited to participate in this sarcopenia substudy will be randomly selected from all 3 study arms. †† Muscle Mass = Dual X-Ray Absorptiometry (DXA) and Magnetic Resonance Imaging (MRI); = JAMAR Hydraulic hand dynamometer.

The study schedule of enrolment, interventions, assessments, and visits for participants is presented in Fig 1.

## Materials and methods

### Trial status

This article outlines the protocol (V7.0 – May 15, 2024). Recruitment for the trial began on September 2, 2022. Recruitment and follow-up will be completed by approximately December 31, 2025. Study results will be made available after data analysis is completed.

### Study design

**Fig 2:**
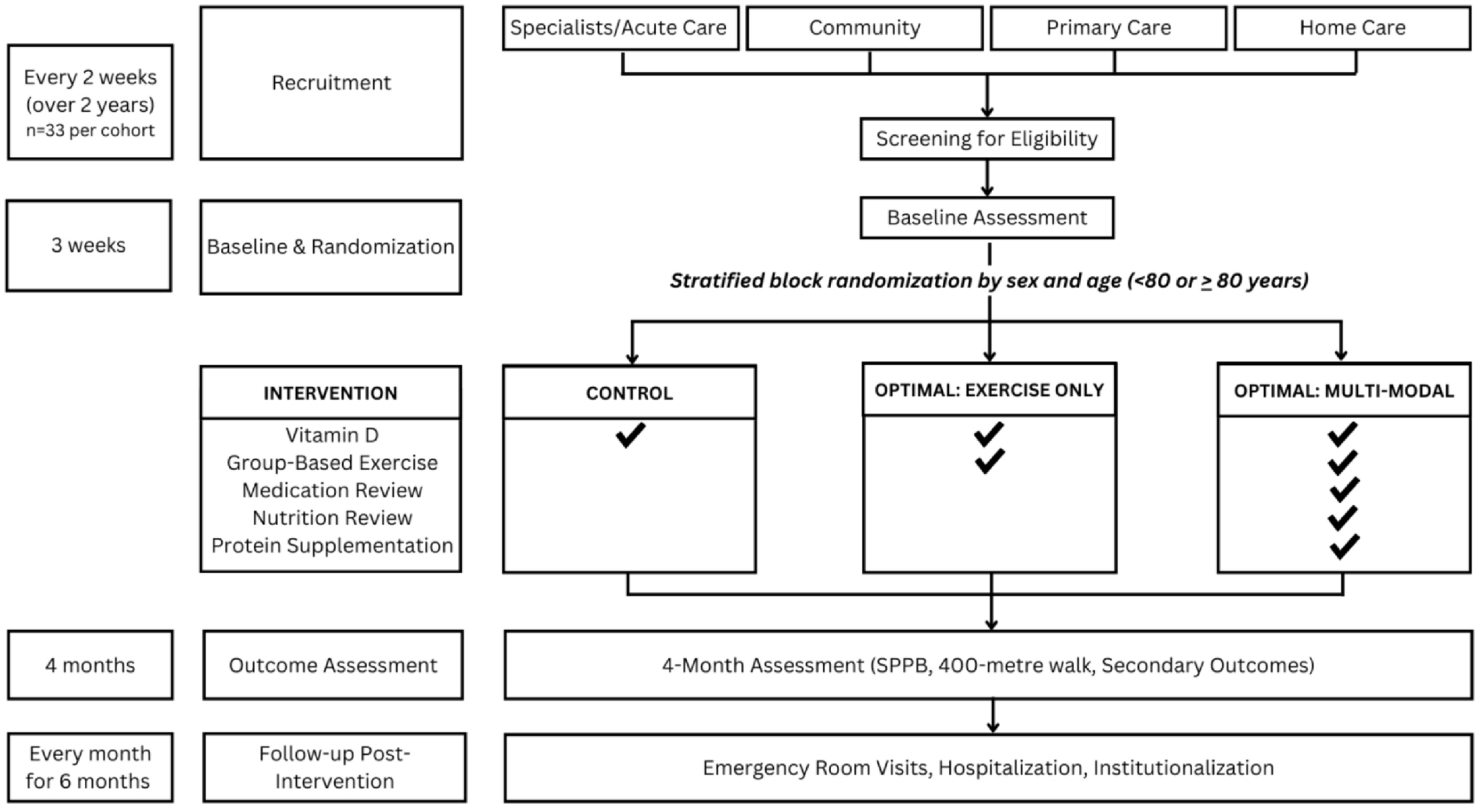
Study overview.

The OPTIMAL Fitness study is a multiarm, parallel-group, partially blinded RCT that will compare the clinical and cost-effectiveness of OPTIMAL Fitness multimodal (group exercise, nutritional intervention (review and coaching/oral nutritional supplementation), medication review/optimization) versus OPTIMAL Fitness exercise only and control. The aim of the study is to determine whether OPTIMAL Fitness exercise or OPTIMAL Fitness multimodal interventions improve physical function versus control in community-dwelling older adults who are prefrail or frail. The main group comparisons will occur at four months. The trial has been registered with ClinicalTrials.gov ID NCT03824106. This protocol manuscript has been formatted according to the Structured Study Protocol Template [28], which embeds the 51 SPIRIT headings and item identifiers within the protocol itself and includes the standardized SPIRIT figure (schedule of enrolment, interventions, and assessments) [29]. The general study design is outlined in Fig 2.

### Study setting {9}

The study takes place in a large, metropolitan area in Southern Ontario, Canada. The recruitment settings will occur within acute care, primary care, rehabilitation units, the community, home care, and external specialist outpatient clinics. The intervention of the OPTIMAL Fitness multimodal and exercise-only arms will occur at the YMCA of Hamilton|Burlington|Brantford, which are accessible locations with access to transportation. The outcome assessments will occur at the YMCA of Hamilton|Burlington|Brantford and via telephone for all three arms. The central site is affiliated with an academic health science centre with several hospital sites serving a catchment area of more than 2.3 million people [30].

### Eligibility criteria {10}

Participants will be included if they are (1) ≥ 65 years of age, (2) able to ambulate 25 meters with or without a walking aid, (3) prefrail or frail (score of ≥2 FRAIL Scale) [31]), and (4) deemed safe to exercise and take oral protein/nutritional supplements (as determined by the referring clinician or primary care provider). Participants will be excluded for the following reasons: 1) unable to speak or understand English (2) currently attending a group exercise program (3) currently in a drug optimization study/program (4) currently taking oral protein/nutritional supplements daily (5) cognitive impairment that prevents them from following two-step commands (6) unstable angina or unstable heart failure; 7) terminal illness; 8) receiving palliative/end-of-life care; (9) travel/commitments that would involve missing >20% of the intervention phase; and (10) other household members enrolled in the study.

### Ethics approval and consent to participate {24}

This study was approved by the Hamilton Integrated Research Ethics Board (#5500).

### Who will obtain informed consent? {26a}

Referring clinicians will obtain initial verbal consent from interested participants, screen for eligibility, and complete the screening intake form, which is submitted to research staff. Interested individuals in the community may also self-refer by contacting the Geras research staff (phone number will be provided on advertising materials). Research staff will contact interested participants over the telephone to confirm eligibility, provide more information, and obtain informed verbal consent. If the participant agrees to participate in the trial, they must provide written consent by signing and dating two copies of the consent form (paper or electronic), which will also be dated and signed by the research staff (one original copy will be given to the participant, and the other copy will be filed by the research staff).

### Study arms

The participants are randomly assigned to one of three arms (control, OPTIMAL Fitness exercise only, OPTIMAL Fitness multimodal). All participants will receive equal amounts of contact for outcome assessments and study adherence by research personnel (e.g., monthly phone calls, outcome assessments). Table 1 provides a summary of the components of each study arm.

**Table 1:** Summary of Interventions by Study Arm.

Intervention Component	Description and dose	ARM 1: Control	ARM 2: OPTIMAL Fitness Exercise only	ARM 3: OPTIMAL Fitness Multimodal
VITAMIN D3	1000 IU/per day	X	X	X
EXERCISE	Group exercise: twice weekly, one hour per class, for four months at the YMCA. Supplemental home exercise: Total of 60 min/week		X	X
MEDICATION REVIEW/ OPTIMIZATION	Nurse assessment: medical and medication history, health teaching/review of any changes; 1–3 appointments, as needed. Pharmacist review using START/STOPP criteria. Recommendations sent to primary care provider and/or community pharmacist.			X
NUTRITIONAL REVIEW	Phone meeting (within first two weeks) with trained research assistant about healthy eating and increasing protein. Review of dietary protein log; Calculation of additional protein supplementation (daily target = 1.2 grams of protein per kg of body weight)			X
ORAL NUTRITIONAL SUPPLEMENTS	One or two oral nutritional supplements/daily (shipped directly to participant) for four months. Each supplement contains 225kcal, 11 grams protein			X

### Explanation for the choice of comparators {6b}

Participants randomized to the control arm will not receive any of the exercise, nutritional, or medication interventions. Participants in all arms, including the control arms, will receive vitamin D3 1000 IU/daily [32]. Vitamin D3 is a low-cost, logistically simple intervention that is recommended for the management of patients with frailty. Furthermore, many older adults are already taking vitamin D3 routinely. Individuals already taking vitamin D3 (1000 IU/or greater) will continue with prescribed treatments.

*Rationale/Evidence:* Lower levels of vitamin D are consistently associated with lower muscle mass and physical function (including handgrip strength test and SPPB) [33–35]. Meta-analyses [36] have demonstrated a positive effect of daily vitamin D supplementation on muscle strength and balance in older adults, an effect influenced by baseline vitamin D deficiency [37,38]. There is good evidence that vitamin D3 supplementation preserves muscle strength and functional ability in high-risk groups, such as older adults with frailty [36,39]*.*

### Description of interventions {11a}

#### OPTIMAL fitness exercise only.

The OPTIMAL Fitness exercise curriculum was developed by a physiotherapist and interdisciplinary team (rehabilitation, geriatric medicine fitness professionals), in conjunction with OPTIMAL Fitness investigators and collaborators (exercise physiologists, geriatricians, occupational therapists, and YMCA trainers), all of whom have expertise in managing older adults living with frailty and are based on prior randomized controlled trial evidence that exercises reduce falls and improve function [36].

*Group Exercise:* The group exercise intervention will be delivered at local YMCA sites by trained instructors and supported by a consultant physiotherapist. Exercises are individually tailored to the individual capabilities of the participants and reassessed monthly by the physiotherapist. YMCA instructors are provided with a structured curriculum and have undergone additional training on working with older adults living with frailty (www.gerascentre.ca).

The participants will attend a group exercise class twice weekly (one hour per class) for four months. The curriculum will emphasize functional movements specifically designed for older adults with frailty and mobility challenges, including modifications for canes/walkers. The exercise instructors were trained on ways to safely modify the exercises to meet the participants’ abilities and needs. The curriculum promotes concurrent training that is safe, progressive, and evidence-based and includes cardiovascular fitness (15 minutes), balance training (20 minutes), strength (15 minutes), and flexibility (5 minutes) [40] (each component is described in Table 2).

**Table 2:** OPTIMAL Fitness Exercise Components.

Exercise Component	Description
Warm-up (5 min)	The purpose of the warm-up is to engage in a general circulatory warm-up prior to beginning the exercise class. Participants complete general gross motor movements to slowly increase their heart rates to prepare them for the remainder of the class. These movements include seated or standing exercises such as kicking forward, arm lifts, ankle plantar/dorsiflexion, arm circles, marching on the spot, knee lifts, heel taps, side stepping, bottom kicks, and side leg lifts.
Cardiovascular (15 min)	Participants progress toward 15 minutes of walking activities with Nordic walking poles by the end of the intervention. The aim of the cardiovascular training is to work at a rate of perceived exertion of light to moderate in the first 8 weeks (2–3 on a 10-point scale), progressing to moderate to vigorous by the end of the program (5–6 on a 10-point scale). Activities include walking in a circle around the exercise room, walking around obstacles, and walking with larger steps.
Balance training (20 min)	Participants engage in challenging balance exercises. Participants begin with static balance exercises (e.g., standing with feet together, single leg stance, tandem stance) and progress toward more dynamic exercises (e.g., weight shifting, stepping around or over objects, stepping to numbers on a clock) throughout the 6 months of the program. Participants are instructed that they should feel challenged by the balance exercises (e.g., they should feel off balance while completing them), but it should still feel safe to complete them.
Functional strength training (15 min)	Participants complete functional strength training exercises mainly focused on the lower extremity. Exercises include activities like sit-to-stand, squats, lunges, calf raises, sideways leg lifts, pick-ups (modified deadlifts), and a floor transfer. For the upper body, exercises included wall push-ups, seated shoulder presses, triceps kickbacks, lateral raises, and seated rows. In line with previous recommendations regarding resistance training for older adults living with frailty [41], participants initially begin with more of an endurance dosage followed by a strength prescription. In the first 4 weeks, the target is for participants to complete 2 sets of each exercise with 10–15 repetitions. In the next phase, participants are encouraged to complete 3 sets of each exercise with 8–12 repetitions (weeks 5–8), and in the final 8 weeks, complete 3 sets with 6–8 repetitions. To progressively increase the intensity of the exercise participants were instructed that the last few repetitions should be challenging.
Cool down and flexibility (5 min)	Participants are guided in a general circulatory warm-down, stretching, and deep breathing. Stretches included those for the chest, hamstrings, calves, gluteal muscles, hip flexors, and quadriceps.

*Supplemental Home Exercise*: The physiotherapist will also perform a one-on-one assessment with each participant at baseline and provide them with a supplemental home exercise program. The participants will be provided with a tailored home-exercise program developed by a physiotherapist to gain an additional hour of physical activity (e.g., 3 sessions of 20 minutes). The home exercise program consists of 5 minutes of warm-up and cardiovascular training (e.g., seated marching), one balance exercise, and two functional strength exercises, followed by 5 minutes of cool down and stretching. Home exercises are modified and tailored every month by the physiotherapist. The study physiotherapist will attend the YMCA exercise class monthly to reevaluate the home-based exercises to ensure that the exercises are at the appropriate level of challenge and to troubleshoot any problems.

*Rationale/Evidence:* Concurrent training (i.e., combined strength and endurance training to increase both aerobic capacity and maximal strength simultaneously) performed at a moderate weekly frequency (i.e., two times per week) may promote marked gains in muscle hypertrophy, strength, and power in older adults living with frailty [42]. Adherence may also be optimized with concurrent training, as it reduces the number of weekly visits to a centre-based program. Our program also incorporates a high challenge to balance, which is a key component of successful exercise programs for vulnerable older adults [40,43] and is most effective for fall prevention [13]. Strong evidence from meta-analyses [40] on exercise to prevent falls in older adults demonstrates that programs that challenge balance and are of a higher dose (at least 180 minutes/week) have greater effects. Thus, our target for the OPTIMAL Fitness program is two centre-based sessions/week (120 minutes) and an additional 60 minutes of home-based exercise.

#### OPTIMAL fitness multimodal.

1. Group and Supplemental Home Exercise
  Group exercise (120 minutes/weekly) and supplemental home exercise (60 minutes/weekly) will be implemented identically to those in Arm 2, as described above and in Table 2.
2. Medication Review/Optimization

The medication optimization intervention is based on a process we have piloted in our FitJoints study [18]. A study pharmacist trained in geriatrics reviews the participant’s medication list and baseline medical history at the beginning of the intervention. The study pharmacist will review 1) the participants’ relevant medical history and 2) medication history, including obtaining participant consent to contact the participant’s pharmacy to obtain a list of current medications. The medical history and medication list will be forwarded to the study pharmacist by the research staff. The study pharmacist will review the medical history and medication list using the Beers’ criteria [44] and the Screening Tool of Older Person's Prescriptions (STOPP) [45]/Screening Tool to Alert to Right Treatment (START) criteria [46] to provide recommendations to the participant's primary care provider and/or community pharmacist. The pharmacist will use their clinical judgement and contact the participant (e.g., telephone, virtual video visit) if any information needs to be clarified prior to making recommendations. Research staff will inform the participant through personalized letters to make an appointment with their family physician to review the medication changes. If medication recommendations are made, research staff will follow up with participants monthly to determine whether the medication recommendations were implemented.

*Rationale/Evidence:* In meta-analyses, clinical consultation, which includes the use of the Beers’ criteria and STOPP/START, has led to a reduction in inappropriate prescribing [46,47]. Deprescribing appears to be feasible and generally safe, and patient‐specific interventions to reduce polypharmacy may improve longevity and reduce adverse events [47].

3 Nutritional Review

At baseline, participants complete the Mini Nutritional Assessment Tool and are booked for a nutritional review phone meeting with a trained research coordinator. Research personnel conduct a nutritional screening flow developed by the study-registered dietician and review the participant’s diet log within 2 weeks of the start of the intervention to determine dietary protein, caloric, and fluid intake during a typical day based on the John Hopkins Medicine Protein Content of Common Foods guide [48]. The research coordinator calculates the recommended additional oral nutritional supplementation needed to achieve a daily target of 1.2 grams of protein per kg of body weight [49]. The participants are also encouraged to refer to their participant manuals, which include nutritional resources from the registered dietician and to focus on eating meals and snacks with good sources of protein and not using protein supplements as meal replacements. Participants who have additional questions regarding their diet and health are directed to follow-up with their primary care provider.

4 Oral nutritional supplementation

Oral nutritional supplements will be provided to all participants in Arm 3 for four months at the start of the intervention unless contraindicated. A minimum of one oral nutritional supplement will be provided for each day of the study. As piloted in our Virtual Frailty Rehab Study [19], the oral nutritional supplement (each serving) contains 225 kcal and 11–12 grams of protein (Glucerna® Nutritional Drink, Ensure® High Protein). The participants will provide the research coordinator with a diet food log to target 1.2 g protein/kg of body weight per day. On the basis of the participants’ diet, food log and protein target, they could receive up to 2 oral nutritional supplements per day with a meal or within 3 hours of exercise on activity days. Participants will receive Glucerna® if they have a diabetes diagnosis, if recommended by their family physician, or by request. The oral nutritional supplements will be sent to the participant’s home via contactless delivery.

*Rationale/Evidence:* In our network meta-analysis, physical activity plus oral nutritional supplementation was an effective intervention for reducing frailty [15]. Adequate protein impacts muscle protein synthesis [50] strength [51] and physical function [52,53] in frail or sarcopenic older adults, which is enhanced with exercise [52]. Although there is still a lack of evidence regarding the effect of protein supplementation alone on frailty, the few existing studies indicate that protein supplementation may be beneficial, particularly for functional outcomes [54]. In an RCT of older adults with frailty, Kim et al. [53] reported that providing two 200-mL liquid formulas (400 kcal, 25 g of protein, and 9.4 g of essential amino acids) per day for 12 weeks improved physical function compared with that of controls. This included a 1-point difference in the SPPB score (which remained stable in the intervention group and decreased by 12.5% in the control group) and an improvement in the timed up and go test (TUG; 7.2% improvement in the intervention group and 3.4% decline in the control group).

### Criteria for discontinuing or modifying allocated interventions {11b}

For a given trial participant, the assigned study intervention may need to be modified or discontinued by trial investigators if the participant experiences harm, which could include injury, hospitalization, a decline in health or mobility status, which impacts the safety of exercise, or withdrawal of participant consent.

### Strategies to improve adherence to interventions {11c}

Research personnel will use telephone call reminders every 6 weeks to follow-up with participants and ensure participants’ adherence to interventions (i.e., home and group exercises, oral nutritional supplementation, vitamin D, follow-up with family physicians, as appropriate) and assist with challenges pertaining to their intervention. YMCA instructors follow a standardized curriculum and have also been trained in working with older adults living with frailty to encourage safe and progressive exercise participation and foster social connections.

### Outcomes {12}

#### Primary outcome.

The primary outcome is physical function as assessed by the SPPB [55] and the 400m Walk Test [56]. The SPPB has three subtests (each scored from 0–4): standing balance (a hierarchical test where stances are progressively more difficult), gait speed (timed four‐meter walk at usual pace), and chair stand (time to complete five consecutive unassisted chair stands). SPPB total scores range from 0 (worst performance) to 12 (best performance); scores ≤8 are indicative of sarcopenia [13,57] and physical frailty [58,59]. The 400m Walk Test consists of 20 laps of 20 meters each. The participants were asked to walk as fast as possible and are allowed no more than 60 seconds of rest during the test [56]. A longer performance time or inability to complete the 400m Walk Test indicates a higher risk of mortality, mobility limitation and mobility disability [60].

#### Secondary outcomes.

The following secondary outcomes will be collected for all participants (the specific measurement variables and collection schedules are listed in Fig 1): 1) frailty and self-reported sarcopenia; 2) activities of daily living; 3) physical fitness; 4) life space mobility; 5) cognition, mood and quality of life; 6) nutritional status; and 7) healthcare utilization. These outcomes are included based on the framework of patient-reported outcome measures (PROMS) [61]. Sarcopenia parameters (muscle mass) will be quantified through dual-energy X-ray absorptiometry (DXA) and magnetic resonance imaging (MRI). A subset of up to n = 75 participants (randomly selected from all 3 study arms) will be assessed with DXA. A subset of up to n = 36 participants will undergo an MRI scan in addition to DXA. All participants will be screened for sarcopenia at baseline. Physical performance based on the 5-time chair stand test with a score of greater than or equal to 12 seconds is predictive of sarcopenia [62]. Among the 324 participants, up to 75 of them with sarcopenia will undergo additional assessments. In accordance with the 2019 Asian Working Group for Sarcopenia Consensus on Sarcopenia and Treatment, we will assess, confirm and grade the severity of sarcopenia at baseline and 4 months. Once the study has reached the capacity of participants with sarcopenia, requisitions for DXA and MRI will no longer be provided. Muscle strength will be assessed with grip strength and chair stand tests. Muscle function will be quantified by gait speed, SPPB, timed up and go, and 400m walk tests.

The baseline characteristics collected will include age, sex, gender (measured by the Physical Self-Attribute Questionnaire) [63], sociodemographic descriptors, chronic conditions, medication use (medication # [total, started, stopped], supplement and vitamin/mineral use, 25(OH)D vitamin D level (mmol/L), and participation in other activation/social programs. After the intervention phase, we will provide all participants with a participation satisfaction survey designed to facilitate a conversation about individual motivators and barriers to participation and attitudes and behaviours surrounding the frailty rehabilitation program.

### Sample size {14}

We calculated the sample size based on our primary outcome of physical function. Our sample size parameters were informed by a study we did with a similar cohort of older adults who participated in an aerobic dance program twice weekly for 12 weeks (GERAS DANCE). Participants who had baseline SPPB scores ≤8 (i.e., indicative of sarcopenia and physical frailty) had a mean change in SPPB total scores of 1.45 (SD 1.97, p < 0.001) between baseline and 12 weeks [64,65]. Previous validation studies indicate that a minimal clinically important difference is an increase of 0.3–0.8 points for SPPB and 0.03–0.05 m/s for the 4 m gait speed. Substantial clinical change is considered an increase of 0.4–1.5 points for SPPB and 0.08 m/s for the 4 m gait speed [64]. The minimal clinically important difference for the 400m Walk Test is 20–30 seconds [64]. Substantial clinical change is considered 50–60 seconds for the 400m Walk Test [64]. We calculated the required sample size for each outcome individually. To ensure that the study is adequately powered to detect a meaningful effect on both outcomes, we adopted the larger of the two calculated sample sizes (SPPBs) as the final sample size for the trial. We conservatively expect at least a 1-point difference in SPPB total scores (SD = 2.0) between the intervention and control arms (multimodal vs control and exercise only vs control). Factoring a drop-out rate of up to 20%, with α = 0.05 and 1-β = 0.9, we need a total sample size of N = 324 (108 per arm).

### Recruitment {15}

We anticipate a randomization rate of 1 enrolled participant per 6 screened and a target recruitment of approximately 3–4 participants/week, based on other studies with older adults living with frailty [18–20,65]. Rolling recruitment will occur over two years, with approximately 10 cohorts of 33 participants (approximately 11 participants per arm) being enrolled across several YMCA sites (Fig 2). Our study team will liaise and advertise the study across our clinical networks with referral sources, including specialist outpatient clinics, acute care, rehabilitation units, and primary care. The study will also be advertised via social media, traditional media, and via our YMCA partners and other community agencies. Interested individuals in the community may self-refer by calling research staff (phone numbers will be provided on advertising materials). Telephone prescreening by the Geras study staff will assist in identifying individuals prior to an eligibility assessment conducted via telephone.

### Sequence generation {16a}

An independent statistician will prepare a computer-generated allocation sequence via SAS 9.4 software [66]. Stratified block randomization (1:1:1 allocation) will be used to randomly allocate eligible and consenting participants to one of three groups (the control group, OPTIMAL Fitness exercise, and OPTIMAL Fitness multimodal). Participants will be stratified by sex and age (<80 years or ≥80 years) with a block size of 3 within each stratum.

### Concealment mechanism {16b}

To conceal the sequence, only a researcher who is not involved in the study will have a computer-generated allocation list. The list will be uploaded into the Research Electronic Data Capture (REDCap) randomization module.

### Implementation {16c}

**Fig 3:**
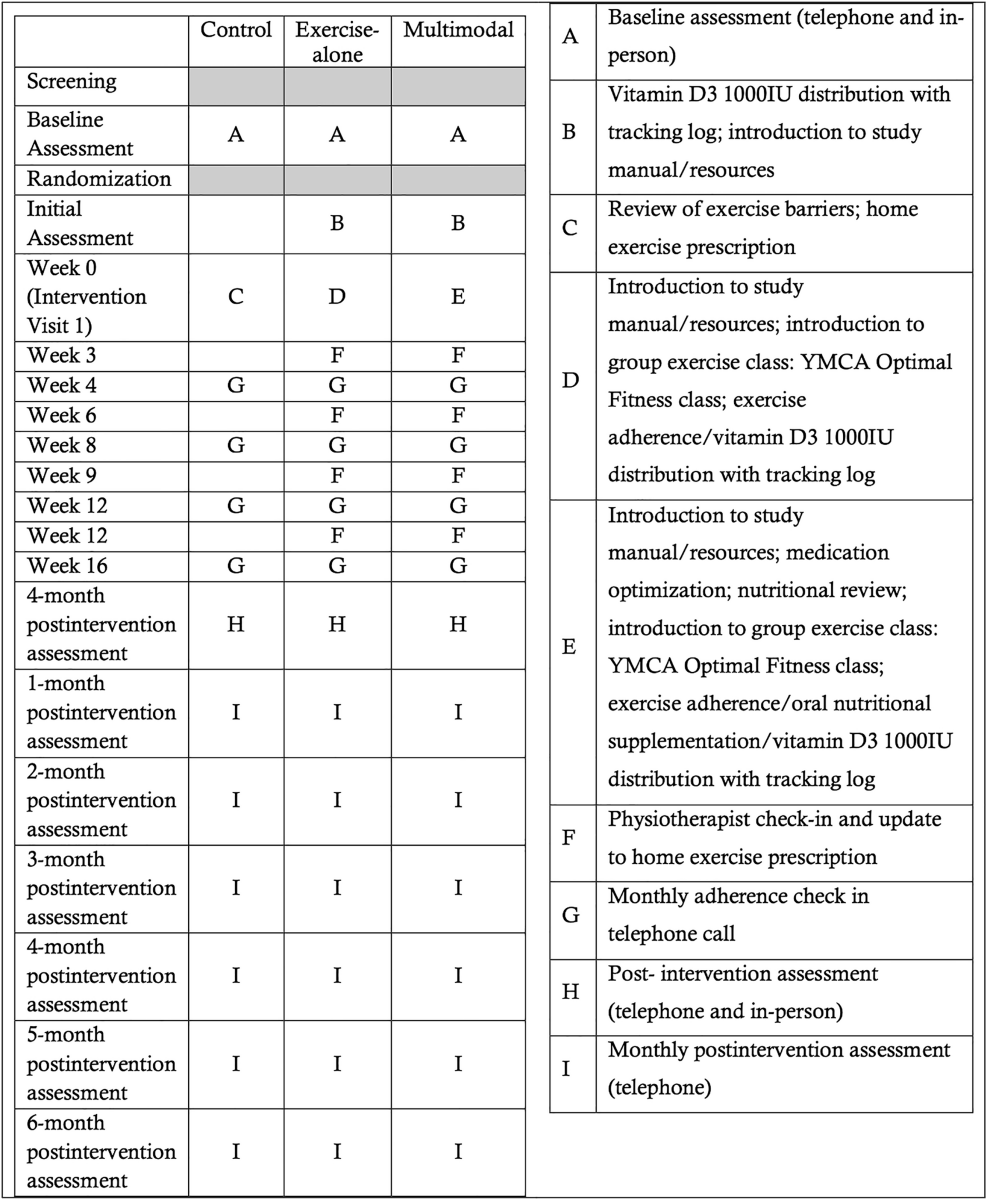
Study intervention and outcome assessments.

Once the study research assistant confirms the participant’s eligibility and obtains informed written consent, the research assistant will submit the eligibility form, consent form, and participant contact form to a team member (who is not part of the study) who will randomize the participant via REDCap. After providing consent, a blinded outcome assessor will contact the participants to set up a telephone appointment and perform an in-person baseline assessment at the YMCA. After the baseline assessment, the research staff will randomize participants within one week of their baseline assessment and inform the participants of their study group. During the first session, participants in the OPTIMAL Fitness exercise only and multimodal groups will receive an intervention-specific study manual, and instructors will provide an orientation for participants so that they are aware of the trial procedures that will follow. A detailed description of the outcome assessments and intervention can be found in Fig 3.

### Who will be blinded {17a}

Outcome assessors, data entry personnel, data analysts who perform the final data analysis, the investigative team, and members of the steering committee will be blinded to the intervention assignments. The participants will also be blinded at the baseline assessment. Due to the nature of the intervention, research assistants, study intervention personnel (pharmacists, instructors) and participants will not be blinded.

### Plans for assessment and collection of outcomes {18a}

The schedule for the study assessments and collection of outcomes is summarized in Fig 1. Blinded assessors will receive training in study procedures and data collection tools. Primary and secondary outcomes will be measured in person at baseline and 4 months. Outcome assessments will take place at a local YMCA site and via telephone. Healthcare utilization and medication/supplement use will be assessed through phone calls by research personnel at baseline, at 4 months, and on a monthly basis for 6 months following the intervention. Participants participating in the sarcopenia substudy will be booked to undergo DXA and MRI within a week of their baseline/4-month post-assessments. A description of the study instruments, along with their reliability and validity, is provided in Table 3.

**Table 3:** Reliability and Validity of Outcomes.

Outcome	Description	Reliability	Validity	Reference
Primary Outcome: Physical Function				
Short Physical Performance Battery (SPPB)	The Short Physical Performance Battery (SPPB) is a tiered evaluation including standing balance, a 4-meter walk, and 5-repeated chair stands. Total scores on the SPPB scale from 0 to 12, with scores below 8 indicating poor physical performance. A significant and clinically meaningful shift in the overall SPPB score is considered to be 1.3 points.	Intraclass correlation coefficient (ICC) = 0.89	Spearman correlation coefficient between SPPB and Barthel Index = 0.853	R: [67] V: [68]
400m walk test	The 400m walk test is a measure to assess physical function and frailty in older adults. The participant walks 400m at their own pace using a walking aid if needed while being timed. Faster times indicate better physical function and lower frailty, while slower times suggest poorer function and higher frailty.	Kappa coefficient = 0.63	Spearman correlation coefficient between 4m and 400m walking speed test = 0.93	R,V: [69]
Secondary Outcomes				
Geras Fit-Frailty App [70]	The Geras Fit-Frailty App is a multidimensional frailty assessment that includes physical, cognitive and psychosocial components – and scored utilizing the Cumulative Deficit Frailty index model. It was developed using the items from our CaMos Frailty Index with an expansion to include other items relevant to measuring multidimensional frailty and sarcopenia. The CaMos Frailty Index [70] comprises 30 items to quantify frailty. It correlates frailty levels with fracture risk, independently of age. Findings suggest that frailty, identified through the index, significantly predicts fracture occurrence over a 10-year period.	No Reported Value	ICC between Fit-Frailty App and Frailty Index-Comprehensive Geriatric Assessment (FI-CGA)=0.65	V: [7, 70]
Self-reported sarcopenia: SARC-F	The SARC-F (Strength, Assistance in walking, Rising from a chair, Climbing stairs, and Falls) questionnaire is a quick screening tool for sarcopenia and physical function. It assesses strength, mobility, and fall risk, predicting hospitalization and mortality in older adults and those with cardiovascular disease.	Cronbach’s alpha = 0.84	Receiver-operating characteristic (ROC) area under the curve (AUC) between EWGSOP2 and SARC-F = 0.64	R,V: [71]
Katz Activities of Daily Living (ADL)	The Katz Activities of Daily Living (ADL) scale evaluates six key functions: bathing, dressing, toileting, transferring, feeding, and continence to determine loss of functions in order of complexity. The measure is utilized for objective evaluations of daily living capabilities.	ICC = 0.999	Spearman correlation coefficient between Katz ADL and Barthel Index = 0.988	R,V: [72]
Lawton Instrumental Activities of Daily Living	The Lawton Instrumental Activities of Daily Living (IADL) Scale evaluates an individual's capability in tasks across eight domains, with a brief administration time of 10–15 minutes. It serves as a potential indicator of early functional decline.	Cronbach’s alpha = 0.94	Spearman correlation coefficient between Lawton ADL Scale and Barthel Index, SF-12 PCS, WOMAC SF and QuickDASH = all correlation coefficients > 0. 40	R,V: [73]
Life Space Questionnaire (LSQ)	The Life Space Questionnaire (LSQ) measures a person's mobility and activity range over a specified time, typically three days to two weeks.	Kappa coefficient = 0.80	Spearman correlation coefficient dictates significant association between LSQ and Performance-Oriented Mobility Assessment (POMA)	R,V: [74]
Montreal Cognitive Assessment (MoCA)	The Montreal Cognitive Assessment (MoCA) is a dementia screening tool. It consists of 30 points, with sections covering orientation, memory, language, mental control, visuospatial skills, and executive abilities. Scores range from 0 to 30, with 26 or higher indicating normal cognitive function.	Test/retest reliability Spearman correlation coefficient = 0.92	Spearman correlation coefficient between MoCa and Mini-Mental State Examination (MMSE) = 0.66	R: [75] V: [76]
Geriatric Depression Scale (GDS-SF)	The GDS-short is a screening tool used to identify depressive symptoms in older adults. The GDS includes 15-items scored dichotomously. Total score is calculated as by tallying the “yes” responses [total score range: 0–15]. Total scores >5 are suggestive of depression.	Cronbach’s alpha = 0.92	Spearman correlation coefficient between GDS-SF and GDS-30 = 0.96	R: [77] V: [78]
Icon Falls Efficacy Scale (ICON-FES)	The Falls Efficacy Scale-International (FES-I) is a tool used to assess an individual's fear of falling across 16 social and physical activities, both indoors and outdoors. It gauges the individual's perceived confidence in carrying out daily tasks without the fear of falling.	Cronbach’s alpha = 0.96	Spearman correlation coefficient between ICON-FES and FES-I = 0.742	R,V: [79]
Health-related Quality of Life (QOL): EQ-5D-5L	The EQ-5D-5L is a self-assessment tool for health-related quality of life, consisting of five dimensions: mobility, self-care, usual activities, pain/discomfort, and anxiety/depression. Each dimension has five levels of problem severity.	ICC = 0.85	Spearman correlation coefficient between QOL: EQ-5D-5L and Visual Analogue Scale (r = 0.772) and 5-point health status scale (r = 0.596)	R,V: [80]
Mini Nutritional Assessment (MNA)	The Mini Nutritional Assessment (MNA) is a tool for quickly assessing nutritional status in elderly individuals. Individuals are categorized into three groups: ‘malnourished’ (<17), ‘at risk of malnutrition’ (17–23.5), and ‘normal nutritional status’ (≥ 24), with a maximum score of 30 points.	ICC = 0.89	ROC AUC between MNA and HemoCue Analyzer 301 for hemoglobin (AUC = 0.845) and Hamwi’s equation for percent of ideal body weight (IBW) (AUC = 0.90)	R: [81] V: [82]
Modified Rating of Perceived Exertion (RPE) Scale^||^	The modified Borg CR10 RPE scale, ranging from 0 to 10, is a tool to measure perceived exertion during exercise based on breathlessness. It is useful for older adults, providing a subjective and reliable way to gauge exertion levels, tailored to their unique physiological responses.	ICC = 0.88	No Reported Value	R: [83]
Healthcare Use (incident)^¶^	Number of visits to primary care provider, walk-in clinic, specialist visits, physiotherapist, urgent care, emergency department; hospitalizations are all self-reported. Collect past 3 months at baseline assessment.	No Reported Value	No Reported Value	[84]
Self-reported falls (incident)	Self-reporting falls tracks the incidence of falls in older adult participants. Participant’s report any falls they experience during a specified period.	No Reported Value	No Reported Value	[85]
Muscle (subset only)††				
Muscle mass: Dual X-Ray Absorptiometry^††^	Dual X-Ray Absorptiometry (DXA) is a widely used method for assessing muscle mass in older adults. It utilizes noninvasive low-dose X-rays to provide accurate measurements of muscle.	ICC = 0.995	Spearman correlation coefficient between DXA and MRI r = 0.94	R: [86] V: [87]
Muscle mass: Magnetic Resonance Imaging^††^	Magnetic Resonance Imaging (MRI) offers a detailed and precise assessment of muscle mass in older adults. MRI uses magnetic fields and radio waves to generate images of the body's soft tissues, including muscles.	ICC = 0.996	Pearson correlation coefficient between MRI and computed tomography (CT) scan = 0.995	R,V: [88]
Muscle strength: handgrip dynamometer [kg] ^=^	The Jamar Hydraulic Hand Dynamometer measures hand and forearm muscle strength through isometric force and peak strength assessment.	ICC = 0.98	Pearson correlation coefficient between Jamar and Dexter dynamometers = 0.99	R: [89] V: [90]
Muscle strength: Timed Up and Go (TUG)	The Timed Up and Go (TUG) test assesses muscle strength and provides an evaluation of lower extremity function. It quantifies mobility by timing the process of standing up from a standard chair, walking a three-meter linear course, turning, and sitting back down.	ICC = 0.96	Pearson correlation coefficient between TUG and Berg Balance Scale = −0.81	R,V: [91]

R: Reliability, V: Validity.

### Plans to promote participant retention and complete follow-up {18b}

Adherence will be measured by a daily self-reported logbook developed specifically for the OPTIMAL Fitness trial to track adherence to home and group exercises, nutrition (oral nutritional supplements and vitamin D), medications, and falls/injuries. The participant logbooks will be collected during the post assessment by the research staff. Study personnel will conduct monthly check-ins by phone to monitor adherence and adverse events and to assess and assist with challenges pertaining to their assigned intervention.

### Data management {19}

Study data will be entered by data entry personnel via REDCap electronic data capture tools. The study database will be password protected and kept on a secure network system. All data entered in REDCap will be audited by a research assistant to assess accuracy and missing data. Only the research team and biostatistician will have access to the final trial database.

### Confidentiality {27}

All the data collected, shared, and maintained will be deidentified to protect confidentiality before, during, and after the trial. Zoom for Healthcare will be used to deliver the nutritional review nutrition component of the intervention. Zoom for Healthcare is HIPAA/PIPEDA compliant. It is security compliant and includes 256-bit Advanced Encryption Standard (AES) encryption, password protection, and waiting room features to ensure the participant’s privacy of information.

### Statistical methods for primary and secondary outcomes {20a}

The study statistician will analyze the study data and report it in accordance with the CONSORT criteria after data collection is completed [92]. A generalized linear model analysis will be used to compare differences between the intervention groups (multimodal vs control and exercise alone vs control) and the control group for both primary and secondary outcomes. The link function in the generalized linear model will depend on the outcome type. For continuous outcomes, we will use an identity link, and for binary outcomes, we will use a logit link. Tukey’s honestly significant difference will be used to analyze the SPPB and 400m Walk Test results. The primary comparisons will be after 4 months. Stratification will be used to increase the precision of the overall estimates in the ANOVA, leading to more precise estimates of the overall treatment effect. We will report between-group differences in means or percentages with 95% confidence intervals at the 4-month post-assessment. All analyses will be an intention to treat.

Economic analysis: A trial-based economic evaluation will be conducted to compare the costs and quality-adjusted life years (QALYs) between the treatment arms. The study conduct and reporting will follow Canadian [93] and international [94–96] guidelines for economic evaluations of healthcare programs. The cost of developing and administering the OPTIMAL Fitness exercise and the OPTIMAL Fitness multimodal interventions will be captured as part of the trial. Healthcare resource utilization data (e.g., hospitalization, emergency department visits, physician visits, and visits to other healthcare professionals) will be collected at baseline, 4 months, and each month post-intervention for 6 months via a short-economic questionnaire that will be developed for the study. Health utility scores derived from the EQ-5D-5L questionnaire [97] collected at baseline and 4 months will be weighted by time spent in health states using an area-under-the-curve approach to calculate QALYs. To address sampling uncertainty, bootstrap techniques [98] will be used, and cost-effectiveness acceptability curves (CEACs) [99] will present the probability of the intervention being cost-effective at different willingness-to-pay thresholds (e.g., $50,000/QALY gained; $100,000/QALY gained). All analyses will be conducted via SAS.

### Interim analyses {21b}

There will be no interim analyses.

### Methods for additional analyses (e.g., subgroup analyses) {20b}

Sensitivity analysis will be performed to examine the per-protocol cohort (participants who completed the trial) and the influence of covariates on our dependent variables, including adherence. We will use a generalized linear model analysis to determine the impact of both stratified variables (age and sex) on the outcome measures. On the basis of the ICEMAN criteria in a randomized controlled trial, we hypothesize that there will be a greater change in the primary outcomes for both women (compared with men) and older adults (compared with younger individuals) [100]. This direction is based on biological evidence [101,102]. In addition, we will also compare differences in both primary and secondary outcomes between the multimodal and exercise alone groups.

### Methods in analysis to handle protocol nonadherence and any statistical methods to handle missing data {20c}

To handle missing data from the intention-to-treat perspective, we will conduct multiple imputation using a Markov chain Monte Carlo method that includes 10 imputed datasets [103]. For sensitivity analysis, a per-protocol analysis will also be performed, including only participants who complete 75% of the intervention. The 75% adherence was selected given that it is a feasible, realistic expectation that is consistent with prior studies [104].

### Ancillary and posttrial care {30}

In the case of research-related side effects or injury as a direct result of taking part in this study, participants will be referred for appropriate medical care. There are no other plans for additional health care or compensation.

### Composition of the coordinating centre and trial steering committee {5d}

The coordinating centre for the study is at the Geras Centre for Aging Research, Hamilton Health Sciences. The study research assistants will be responsible for submitting and maintaining REB documents, liaising with clinical partners for recruitment, enrolling participants, receiving and storing consent forms, monitoring adherence and adverse events, overseeing data collection and entry, and all other trial day-to-day operations. To provide overall supervision of the trial, the study steering committee will meet every 6 months or more frequently, as needed.

### Composition of the data monitoring committee, its role and reporting structure {21a}

The Data and Safety Monitoring Board (DSMB) is an independent multidisciplinary group consisting of a biostatistician and clinicians who, collectively, have experience in the management of older adults who are prefrail or frail and in the conduct and monitoring of randomized clinical trials. For each DSMB meeting, Open and Closed Reports will be provided. Open Reports, available to all individuals who attend the DSMB meeting, will include data on recruitment and baseline characteristics and pooled data on eligibility violations, completeness of follow-up, and compliance. Closed Reports, available only to those attending the Closed Sessions of the DSMB meeting, will include analyses of AEs and symptom severity and Open Report analyses that are displayed by the intervention group.

### Adverse event reporting and harms {22}

Study personnel will conduct monthly check-ins by phone to monitor for adverse events. The participants will also be instructed to contact the study coordinator if they experience any unfavourable signs or symptoms. Adverse events or harm from any source will be reported to the research team and recorded in a structured form. Any serious adverse events will be reported to the Research Ethics Board within 24 hours. The independent DSMB described above will review safety data from the trial and advise the investigators and the Steering Committee on the future management of the trial.

### Frequency and plans for auditing trial conduct {23}

There are no plans for auditing to be conducted.

## Discussion

Previous studies have examined the effect of a multimodal intervention to reduce frailty; however, the trials conducted were limited by small sample sizes and the number of adults with frailty and lacked sufficient power to detect the potential additive effect of a multimodal intervention to treat frailty [15]. Furthermore, the interventions were based on a geriatric/rehabilitation service and were resource intensive. Large clinical trials examining interventions for the treatment of sarcopenia are also lacking; thus, there has been limited evidence to establish recommendations for their use in treating sarcopenia [16].

The OPTIMAL Fitness trial will examine a model of frailty rehabilitation that could be implemented within the community. Our pilot research has demonstrated the feasibility of community-based frailty interventions in Canada without the use of specialized assessment teams [105]. Given the high co-occurrence of frailty and cognitive impairment [105], we have demonstrated that individuals with mild to moderate cognitive decline can successfully participate in group exercise [106]. Given our rapidly aging population, this trial will provide valuable insight into the clinical effectiveness and feasibility of rehabilitation services for vulnerable older adults using bundled care with socialization, exercise, nutrition, and medication support. If the frailty rehabilitation program is found to be effective within our setting, the next phase is to consider the generalizability of our approach, i.e., how this model could be applied within communities of varied sizes, demographic compositions, and geographical locations.

### Strengths and limitations

The strengths of our proposed study include (1) a large sample size that is sufficient to attain adequate power to detect a difference in outcomes and robust conclusions may be drawn; (2) a study design that allows us to consider the additive effects of multimodal interventions that include other important components for comprehensive frailty and sarcopenia management (e.g., socialization, nutrition, protein, and medication support) in addition to exercise; (3) rigorous methodological safeguards to avoid potential bias, including a centralized randomization system, assessment of baseline measures prior to randomization, and blinding outcome assessors, study biostatistician, the investigative team, and the steering committee to intervention assignments; and (4) engagement of all key stakeholders in the process of implementation, including patients, interdisciplinary healthcare teams, researchers, and community organizations, as indicated in the Medical Research Council guidance for complex interventions [26]; and (5) although our central site is in a community with an academic health science centre, our community-based model allows the potential for uptake in smaller centres with the ability to incorporate additional consultation via virtual methods. We have recently demonstrated that virtual frailty rehabilitation is a viable approach [19].

The limitations of this study include: (1) the inability to blind research assistants, intervention personnel (pharmacists, instructors), and participants, which may introduce bias; this is mitigated by using objective primary outcomes and blinding outcome assessors and the biostatistician conducting the analyses; (2) the limited follow-up period inherent to the RCT design, which prevents assessment of long-term benefits or harms; (3) exclusion of participants with significant cognitive impairment or those unable to speak English, which may limit generalizability; and (4) the multifaceted nature of the intervention (exercise, nutrition, and medication evaluation), which prevents isolation of the effects of individual components.

### Dissemination plans {31a}

As a CIHR-designated Canadian Research Centre on Aging, the Geras Centre for Aging Research is aligned with the CIHR Strategic Plan 2021–2031 [107] bridging research and implementation to promote knowledge mobilization (KMb) and evidence-based practices in healthcare. Our KMb approach includes engaging stakeholders with lived experience (older adults are part of our advisory board, fostering partnerships with policymakers, healthcare providers, and community organizations, and disseminating findings through multiple channels (e.g., peer-reviewed publications, conference presentations, social media, and local geriatric networks). The research findings will be shared with an extensive network of institutes and centres (e.g., the McMaster Institute for Research on Aging (MIRA)), health professionals, policymakers, educators, community stakeholders (e.g., the Hamilton Council on Aging, YMCA), and participants and their families and/or caregivers.

### Plans for communicating important protocol amendments to relevant parties (e.g., trial participants, ethical committees) {25}

All protocol updates (i.e., changes to eligibility criteria, outcomes, procedures, etc.) will be communicated to relevant parties (i.e., investigators, research teams, participants, etc.) and submitted to the ethics board for review.

## Supporting information

S1 File SPIRIT Checklist. (PDF)

S2 File Protocol. (PDF)

S3 File Fit-Frailty App (OPTIMAL Fitness version). (PDF)

## Abbreviations

CI: confidence interval
CGA: comprehensive geriatric assessment
DSMB: data and safety monitoring board
EWGSOP: European working group on sarcopenia in older people
GEE: generalized estimating equation
HR-QOL: health-related quality of life
OPTIMAL: OPTimizing independence, mobility, and active life
SDOC: sarcopenia definition and outcomes consortium
SPIRIT: standard protocol items: recommendations for interventional trials
SPPB: short performance physical battery
START: screening tool to alert to right treatment
STOPP: screening tool of older person's prescriptions
QALYs: quality-adjusted life years
RCT: randomized controlled trial
REDCap: research electronic data capture
