# Supplementary material for: Study protocol for a multiarm, randomized controlled trial to determine the effectiveness of community-based frailty rehabilitation to improve physical function in older adults: The OPTIMAL Fitness Trial
Source: PLoS One. 2026 Mar 12;21(3):e0343338. doi: 10.1371/journal.pone.0343338 (PMC12981438; doi:10.1371/journal.pone.0343338)
Supplement: S2 File — (PDF) [file pone.0343338.s002.pdf]

Frailty Rehabilitation: A Community-Based Intervention to Promote Healthy Aging

Principal Investigator

Dr. Alexandra Papaioannou, MD, MSc, FRCP(c), FACP

Executive Director, GERAS Centre, Hamilton Health Sciences, St. Peter's Hospital, Hamilton, ON

Professor, McMaster University, Department of Medicine, Division of Geriatric Medicine, Hamilton, ON

GERAS Research Coordinator and Research Assistant *GERAS Centre for Aging Research, Hamilton Health Sciences, St. Peter's Hospital, Hamilton, ON*

Co-Investigators:

Dr. Rick Adachi[1,3]
Dr. Pankaj Eric Bansal[4]
Dr. Pauline Boulos[1]
Dr. Lisa Dolovich[1,5]
Dr. Patricia Hewston[1,3]
Ms. Genevieve Hladysh[6]
Dr. George Ioannidis[1,2]
Dr. Sharon Kaasalainen[7]
Dr. Courtney Kennedy[1,3]
Dr. Justin Lee[1,3]
Dr. Dee Mangin[2,8]
Mr. Shyam Maharaj[4]
Dr. Sharon Marr[1,3]
Dr. Caitlin McArthur[3,9]
Dr. Ahmed Negm[3,10]
Dr. Michael Noseworthy[11,12]
Dr. Alexander Rabinovich[13,14]
Dr. Kenneth Rockwood[15]
Dr. Lehana Thabane[2]
Dr. Jean-Éric Tarride[2]
Dr. Olga Theou[15]

1 McMaster University, Department of Medicine, Hamilton, ON
2 McMaster University, Department of Health Research Methods, Evidence and Impact (HEI), Hamilton, ON
3 GERAS Centre for Aging Research, Hamilton Health Sciences, St. Peter's Hospital, Hamilton, ON
4 Hamilton Health Sciences, Hamilton, ON
5 University of Toronto, Leslie Dan Faculty of Pharmacy, Toronto, ON
6 YMCA of Hamilton, Burlington, Brantford, ON
7 McMaster University, School of Nursing, Hamilton, ON
8 University of Otago, Family Medicine, Christchurch, New Zealand
9 Dalhousie University, School of Physiotherapy, Halifax, NS
10 University of Alberta, Faculty of Rehabilitation Medicine, Edmonton, AB
11 McMaster University, School of Biomedical Engineering, Hamilton, ON
12 McMaster University, Department of Electrical and Computer Engineering, Hamilton, ON
13 McMaster University, Faculty of Health Sciences, Hamilton, ON
14 ArthroBiologix Inc., Hamilton, ON
15 Dalhousie University, Department of Medicine, Halifax, NS

Funding Sources:

CANADIAN INSTITUTES OF HEALTH RESEARCH
HAMILTON ACADEMIC HEALTH SCIENCES ORGANIZATION

1.0. INTRODUCTION

Frailty can be characterized as a state of diminished reserves (energy, physical ability, cognition, health) to the extent that function is reduced and the person is more vulnerable to external stressors (e.g., new medication or urinary tract infection) [1,2]. Our research suggests that approximately 23% of Canadians over age 65 are frail [3] and by age 85 this estimate increases to over 40% [4]. Older adults who are frail are high users of healthcare services [5] and those with moderate or severe frailty have an 8-fold greater relative risk of institutionalization (95% CI= 4.9-15.2) [6]. Thus, within our rapidly aging society, **there is a clear need for feasible, effective and sustainable community-based models to address the enormous problem of managing frailty.**

Two important considerations about frailty from a management perspective are: 1) frailty is **dynamic in nature (i.e., may improve or worsen over time)** [7] and 2) frailty is **reversible or treatable** [8]. Although frailty consensus recommendations [9] suggest treatment with specific modalities (exercise; reduction of polypharmacy; vitamin D3; protein supplementation), there is little evidence regarding whether a targeted, community-based approach can improve frailty or prevent decline and whether additional management approaches are required over and above exercise. Drawing on the principles of a standardized multi-modal approach in **cardiac** [10,11] and **cancer rehabilitation** [12,13], frailty rehabilitation could be an accessible community-based intervention with the **ultimate goal** to improve physical function, reducing frailty, and enabling independence within the community.

<u>**The LIFE trial**</u> which examined physical activity in sedentary adults had a relatively small number of "frail" adults and was focused on walking (up to 150 min/week) with only 10-min strength per session. However, the LIFE trial ***did not meet the minimum criteria for 180 min/week of exercise*** (with a high challenge to balance) recently identified effective for fall prevention [14]. <u>**The Australian FIT trial**</u> [15] a land-mark trial, demonstrated that multi-modal intervention significantly reduces frailty and was the first to demonstrate that treating frailty was a realistic therapeutic goal [8]. This intervention was ***resource intensive and based within a geriatric/rehabilitation service,*** which may not be feasible in many communities across Canada and given the wait-lists. The FIT trial was not able to determine the effects of exercise alone. <u>**The Singapore FIT trial**</u> [16] examined exercise, cognitive training, and nutritional supplementation separately versus multi-modal, however over 70% were pre-frail. This trial was not a model of multi-modal rehabilitation delivered in a community setting and did not include medication review (an important component for comprehensive frailty management). This trial was ***not powered*** to detect statistically significant differences between intervention-groups (i.e. the potential additive effect of the multi-modal intervention over individual interventions), however, a reduction in frailty was observed in the multi-modal intervention group compared to the control group (OR=5.00, 95% CI=1.88-13.3). <u>**Our pilot work**</u> has demonstrated the feasibility of community-based frailty interventions in Canada without the use of specialized assessment teams. With the high co-occurrence of frailty and cognitive impairment [17], we have demonstrated individuals with mild to moderate cognitive decline can successfully participate in group exercise. Thus, our next step is a larger-scale trial to definitively consider the effectiveness of such an approach compared to less intensive interventions. Building upon our previous research, our aim is to consider if frailty rehabilitation is feasible and effective for a large proportion of older adults living with frailty who are community-dwelling (i.e., not housebound and able to follow instructions to

participate in the group). More specifically, this trial is the first of its kind to examine a **model of frailty rehabilitation** (that could be implemented within the community) and extends the previous literature. The frailty rehabilitation study will be publicly known as the OPTIMAL Fitness study to include positive language associated with aging to assist with recruitment of community-dwelling older adults.

Frailty and Sarcopenia: While there is some overlap between sarcopenia and frailty, sarcopenia may be the pre-cursor to frailty. Recent consensus guidelines define sarcopenia as a muscle disease ("muscle failure") with low strength, low muscle quantity and low physical performance (17). According to the European Working Group on Sarcopenia in Older People (EWGSOP), approximately 50 million people currently live with sarcopenia worldwide and is projected to increase to 200 million in the next 40-years (17). Sarcopenia is associated with increased fall-risk, fractures, mobility disability, loss of independence and reduced quality of life. Furthermore, older adults with sarcopenia are 5x more likely to have increased hospital costs compared to those without sarcopenia (OR = 5.70, 95% CI 1.57–20.71). Therefore, optimal care of older adults with sarcopenia is essential to reduce the personal, social and economic costs when untreated (17). Building upon our CIHR RCT, we aim to understand which components of a functional rehabilitation program are essential to change the trajectory of sarcopenia in older adults with sarcopenia.

2.0. OBJECTIVES

<table>
<tr><td>PRIMARY</td><td>In community-dwelling older adults living with frailty and sarcopenia, does a 4-month multi-modal intervention (Arm 3) improve physical function (Short Physical Performance Battery and 400-m Walk Test) compared with control (Arm 1) and group exercise alone (Arm 2)?</td><td rowspan="4">HYPOTHESES:
- Frailty rehabilitation will achieve the most successful results in frail and sarcopenic cohorts and offer enhanced benefits relative to the other arms. Socialization and exercise only will provide clinically meaningful improvements to physical function and frailty over time, with exercise alone achieving greater benefits than control (usual care).
- Frailty rehabilitation will result in greater reductions in healthcare utilization in frail and sarcopenic older adults relative to the other arms.
- A range of functional abilities and health-related quality of life will be identified in older adults living with frailty.</td></tr>
<tr><td rowspan="3">SECONDARY</td><td>To better understand the functional abilities (frailty; fear of falling; fitness; strength; cognition; health-related quality of life; life space mobility; activities of daily living; depression/mood; nutrition) of older adults living with frailty and sarcopenia and translate the first Canadian model of frailty and sarcopenia rehabilitation and management.</td></tr>
<tr><td>Determine which components of a functional rehabilitation program are essential to change the trajectory of sarcopenia (muscle mass; muscle strength; muscle function).</td></tr>
<tr><td>Determine the feasibility of a functional rehabilitation program (recruitment rate, retention rate, data collection, adherence to all interventions).</td></tr>
</table>

**EXPLORATORY**	Does a 4-month multi-modal intervention (Arm 3) reduce **healthcare utilization (emergency room visits; hospitalization; home care service use; institutionalization)** compared with control (Arm 1) and group exercise alone (Arm 2) during a 6-month follow-up period?	

3.0. THEORETICAL FRAMEWORK

Our frailty rehabilitation program is based on the international consensus guidelines that recommend a **multi-modal approach** to reduce frailty [9] and the **WHO** Public-Health Framework for Healthy Ageing [18] to align community-based health services to meet the complex needs of older adults who are frail. Their call to action is "affordable access to integrated health services that are centered on the needs of older people" [19]. This framework for action is built around the concept of building **intrinsic capacity** (defined as all the physical and mental capacities that an individual can draw on at any point in time) by **promoting capacity-enhancing behaviours** within a supportive environment to **enable functional ability** (defined as health-related attributes that enable people to be and to do what they have reason to value) [19]. Frailty and intrinsic capacity are closely linked constructs given that frailty is the age-related decline of physiological systems, which can profoundly reduce intrinsic capacity [20].

4.0. METHODS

4.1. Design: In this multi-arm RCT, 324 community-dwelling older adults (aged 65+) with frailty and at high risk for mobility disability will be randomized into one of three arms (**control, exercise only, multi-modal rehabilitation**) stratified by sex, age, and location preference. Rolling recruitment will occur with ten cohorts total (2-3 cohorts per site, n=33 participants per cohort), enrolled across the partner YMCA sites. Blinded assessors will assess clinical outcomes at 0 and 4-months, with an additional 6-months of healthcare utilization via telephone follow-up. The multi-modal intervention is adapted from protocols used in our prior pilot studies and designed to ensure safe training components for frail adults with an emphasis on improving functional ability.

4.2. Recruitment Process: Our team (Geras, McMaster University, and Hamilton Health Sciences staff, students/trainees, and volunteers) will collaborate with clinicians (e.g., physicians, nurses, physiotherapists, occupational therapists, case managers) and administrative staff/ services (e.g. HHS Hub) at Hamilton Health Sciences (HHS) (e.g. St. Peter's Hospital, Juravinski Hospital, Hamilton General Hospital), McMaster University, and St. Joseph's Healthcare Hamilton (SJHH) to identify potential participants from a variety of units. Additional sources of referral will be from any healthcare provider, including but not limited to, physicians, nurses, physiotherapists, occupational therapists, case managers, within Southern Ontario (external to HHS, McMaster, and SJHH). If requested/approved, these networks will also be provided with brochures, posters, and other study advertisements for recruitment.

A pre-screening referral form will be completed and sent to the research team to determine eligibility. If the referee is external to HHS/McMaster, the referee will be asked to sign a data transfer agreement prior to using

the pre-screening referral form for sending patient information. Interested individuals in the community may also self-refer by calling or emailing the GERAS Research staff (phone number will be provided on advertising materials) or completing the contact form on the Geras Centre website (mandatory fields include first name, email address or telephone number, and location preference). Telephone pre-screening by the GERAS study staff will assist in identifying individuals prior to a virtual (via telephone) eligibility assessment. During the telephone pre-screening, we will also be asking the participant whether they would prefer a study information package to be mailed to an address of their choice or e-mailed. The study information package includes the following: a one-pager study info sheet and the participant information letter to allow the participant to learn more about the study at their own pace prior to informed consent. Similar studies in primary care with frail elderly report randomization rates of 1 enrolled patient per 6 screened. Our recruitment strategy will include:

- **Acute Care:** Our team will collaborate with clinicians (physicians, nurses, physiotherapists, occupational therapists) in acute care (e.g., at the Juravinski Hospital) to identify potential participants being discharged from medical units under the direction of the unit's director. These individuals will help the team identify and recruit potential participants from acute care by: a) having a member of the research team located at the clinic to directly assist with recruitment (e.g., talking to prospective participants), b) having the clinician discuss and/or distribute advertisement material/study information (e.g., via brochures or smartphrases used in EPIC for patient discharge or visit forms/sheets), or c) asking the clinician to use the Clinical Pre-Screening Referral Form (**Appendix A**; this includes obtaining patient consent for a member of the study team to contact them to arrange a formal study-screening visit via telephone) and send this form to the GERAS research team.
- **Primary Care:** Our team is well connected within primary care (including family medicine leaders Drs. Mangin and Dolovich and family doctor collaborator Dr. Kristof) and several co-Investigators are part of the healthtapestry.ca primary care initiative (Co-led by Dr. Dolovich). Drawing on these networks, we will work with family health teams and general practitioners (GP). For practices with electronic medial records, screening for eligibility is a strategy that has been successfully applied with similarly vulnerable cohorts [21]. Our research team will support family health teams and administer telephone pre-screening. Clinicians may also refer patients directly by completing the Clinical Pre-Screening Referral Form (**Appendix A**) and send to the GERAS research team. **In our region, in a typical family health team (e.g. 4 physicians), there are approximately 6000 patients, with 10% over age 70 (n=600)** (personal communication/query). Recruitment at the clinic may look like: a) having a member of the research team located at the clinic to directly assist with recruitment (e.g., talking to prospective participants), b) having the clinician discuss and/or distribute advertisement material, c) asking to clinician to use the Clinical Pre-Screening Referral Form (Appendix A; this includes obtaining patient consent for a member of the study team to contact them to arrange a formal study-screening visit) and send this form to the GERAS research team, d) having a member of the research team contact patients who have already agreed to be contacted for research studies (e.g., in a research registry at a healthcare organization), and/or e) asking the clinic to perform a database search for potentially eligible participants and subsequently asking the clinic/clinicians to contact/discuss potential study enrollment with these patients (via mail,

in-person, phone etc.). If option E (database search), with the permission of the clinic, the Geras research team may assist the clinic with mailing.

- **Community:** We already have and continue to establish partnerships with other studies (e.g. GERAS DANCE, community centres and organizations (e.g. Alzheimer's Society, Osteoporosis Canada). Within the community, research personnel will utilize user-friendly brochures and attend events to promote the study (e.g., at food drives, community centres, libraries, places of religious worship). Research personnel will also connect with local media outlets to advertise for recruitment (i.e., newspapers, television, radio, etc.). In addition, this study will be advertised on the Voice platform (a web-based self-referral through advertising recruitment pathway). The Voice is a community engagement platform that the McMaster Institute for Research on Aging (MIRA) is licensing from the University of Newcastle. Please see Appendix C for more details on recruitment for this study through the Voice.
- **Home Care:** Older adults receiving homecare represent over 50% of emergency room visits at Hamilton Health Sciences (LHIN Personal Communication). Our team is already embedded with the LHIN and will work with LHIN care coordinators who may identify high-risk clients who are frail yet mobile to attend community classes and benefit from this study.
- **External Specialists within Community:** These individuals will help the team identify and recruit potential participants from their clinics. This may be done by: a) having a member of the research team located at the clinic to directly assist with recruitment (e.g., talking to prospective participants), b) having the clinician discuss and/or distribute advertisement material, c) asking the clinician to use the Clinical Pre-Screening Referral Form (**Appendix A**; this includes obtaining patient consent for a member of the study team to contact them to arrange a formal study-screening visit) and send this form to the GERAS research team, d) having a member of the research team contact patients who have already agreed to be contacted for research studies (e.g., in a research registry at a healthcare organization), and/or e) asking the clinic to perform a database search for potentially eligible participants and subsequently asking the clinic/clinicians to contact/discuss potential study enrollment with these patients (via mail, in-person, phone etc.). If option E (database search), with the permission of the clinic, the Geras research team may assist the clinic with mailing.

***4.3. Intake Appointment*:** All participants will attend an intake appointment where **eligibility screening**, and **informed consent** will be reviewed by the GERAS study staff. Using the recruitment strategy indicated above in our pilot studies, we recruited 3-4 participants/week with frailty and cognitive impairment (GERAS DANCE) and 3-4 participants/month scheduled for hip or knee replacement surgery (Fit Joints). The loss to follow-up was 20% (GERAS DANCE) and 10% (Fit Joints). Within the literature, the Singapore FIT trial [16], which included also pre-frail individuals, had a low drop-out rate of 4-10% (depending on group assignment).

4.4. Eligibility Criteria

Inclusion Criteria:

- Community-dwelling (not LTC) ≥65 years of age;
- Able to ambulate 25 metres with or without a walking aid;

- At high risk for mobility disability/functional limitations as assessed by the John Morley FRAIL scale (score of ≥2);
- Medical clearance from referring clinician, or for self-referrals, medical clearance from family physician to safely participate in exercise and take oral nutritional supplements;
- Can arrange transportation to the YMCA up to 2 times per week;

Exclusion Criteria:

- Unable to speak or understand English;
- Currently attending a group exercise program;
- Currently in a drug optimization study/program;
- Currently taking oral nutritional supplements daily;
- Significant cognitive impairment where they may have difficulty following two step commands in group exercise [22];
- Receiving palliative/end of life care;
- Terminally ill;
- Unstable angina or unstable heart failure;
- Another household member enrolled in the study;
- Unable to attend for more than 20% of trial duration.

Participants are eligible to participate as long as they abide by the current COVID-19 protocols at all study sites at the time of enrollment. During the baseline assessment, all eligible participants will have sarcopenia status assessed, by examining physical performance (through the SPPB), prior to randomization. Scores greater than or equal to 12 seconds for the 5-time chair stand test are predictive of sarcopenia. Participants with sarcopenia will undergo additional outcomes to measure changes in muscle mass and strength (see Table 2 for outcomes). Up to 75 participants with sarcopenia will undergo these additional outcomes at baseline and 4-month time points. Participants who complete the 5-time chair stand test in less than 12 seconds are still eligible to participate, provided they meet the other eligibility criteria (listed above) during screening.

4.5. Randomization: Stratified block randomization (1:1 randomization ratio) with the allocation sequence generated by a computer will be used to randomly allocate participants to their group assignment. Participants will be stratified based on their sex, age (<80 or ≥ 80 years), and location preference. Research staff will obtain informed consent and confirm eligibility and will randomize the participant using Research Electronic Data Capture (REDCap) randomization system, a secure web application for building and managing online surveys and databases. To protect against selection bias, the randomization sequence will be adequately concealed so that investigators/participants are not aware of the upcoming assignment. To protect against ascertainment bias, baseline measures will be assessed **prior** to randomization. Outcome assessors, the study biostatistician, the investigative team, and the steering committee will be blinded to intervention assignments. Research assistants, study intervention personnel (pharmacist, instructors) and participants will not be blinded.

4.6. Interventions: Participants will be randomized to one of three arms (control, group exercise only, multi-modal), with the intervention lasting 4-months (**Figure 1**). Each cohort will have at maximum, 11 people

randomized into each arm for a total of 33 individuals per cohort. **Table 1** summarizes preliminary findings of our network meta-analysis [23]. All participants will receive equal amounts of contact and follow-up by research personnel (e.g., monthly phone-calls, outcome assessments). Participants from all 3 arms will be randomly selected to wear a study ActiGraph accelerometer for the duration of the study, should they consent. The device will collect health information such as raw acceleration data, physical activity intensity, total sleep time, activity bouts, sedentary bouts, sleep efficiency, and energy expenditure, in real time.

Study Orientation: During the first session, participants in the multi-modal and exercise only groups will receive an intervention-specific study manual and instructors will provide an orientation for participants so that they know what to expect from their sessions.

Table 1: Frailty Management – Summary of Evidence for Individual Components

**SOCIAL**	**Rationale:** Social interaction associated with better mental and physical health [24], and evidence suggests that group versus individual engagement may offer unique cognitive benefits for older adults [25] as well as peer reinforcement and encouragement.
**VITAMIN D3**	**Dose/Frequency:** 1000 IU Vitamin D3 daily [26]<br>**Rationale**: Lower levels of Vitamin D levels are consistently associated with lower muscle mass and physical function (including handgrip strength test and SPPB) [27–29]. Meta-analysis [30] demonstrates a positive effect of daily vitamin D supplementation on muscle strength and balance in older adults, an effect influenced by baseline vitamin D deficiency [31,32].
**EXERCISE**	**Dose/Frequency:** A recent meta-analysis suggests 180 min/week of exercise (with a high challenge to balance) is most effective for fall prevention [14]<br>**Rationale:** Combined strength and endurance training (i.e., concurrent training) performed at a moderate weekly frequency (i.e., two times per week) may promote marked gains on muscle hypertrophy, strength and power gains in older adults living with frailty [33]. Balance training is a key component of successful exercise programs for vulnerable older adults [34].
**PROTEIN**	**Dose/Frequency:** As piloted in our Virtual Frailty Rehab study, the oral nutritional supplement (ONS) (each serving) contains 225kcal, and 11-12 grams protein (Glucerna, Ensure). Participants are advised to take this with a meal or within 3 hours of exercise on activity days. Participants will be provided with two ONS each day of the study.<br>**Rationale:** Adequate protein impacts muscle protein synthesis [35] strength [36,37], and physical function [38,39] in frail or sarcopenic older adults that is enhanced with exercise [39]. Older adults with malnutrition or who are at risk of malnutrition are recommended to consume oral nutritional supplements per day containing at least 400kcal and 30 grams of protein [40].
**MEDICATION**	**Approach:** In meta-analyses, improving the appropriate use of polypharmacy in older people can be obtained with use of Beers' criteria and Screening Tool of Older Person's Prescriptions (STOPP)/ Screening Tool to Alert to Right Treatment (START) have demonstrated a reduction in inappropriate prescribing [41].<br>**Rationale**: Deprescribing appears to be feasible and generally safe. Patient-specific interventions to reduce polypharmacy may improve longevity and reduce adverse events [42].

Arm 1: Control:

Participants randomized to the control arm will not receive any of the Frailty Management Interventions (see Table 1).

Arm 2: Group Exercise:

Group Exercise: Participants will attend the exercise program, ***twice-weekly*** (one-hour per class) at a YMCA site for 4-months. In the first 15-minutes, participants will check-in and report any injuries/concerns. The **exercise component** will be an hour of strength/balance exercises and aerobics (walking with activator poles supervised by an instructor) followed by time allotted for participants to **socialize**. The supervised group exercise component will be modeled after existing **LiveWell classes** at the YMCA (e.g., *Balance Plus*, *In Motion*) but with a greater emphasis on functional movements specifically designed for older adults with frailty and mobility challenges, including modifications for canes/walkers. Principles of balance training from the *Weight-bearing Exercise for Better Balance* [WEBB.org.au; used in the Frailty Intervention Trial [8,15]] will be included. Co-Investigator, Dr. Caitlin McArthur, a registered physiotherapist and post-doctoral fellow has tailored the existing YMCA programs to meet the needs of older adults living with frailty based on challenging, safe, progressive evidence-based exercises: 1) resistance 2) balance 3) aerobic 4) static and 5) dynamic stretching (flexibility) in addition to a warm-up and cool-down. **Supplemental Home Exercise*:*** In accordance with recent guidelines to ***achieve 3 hours/week*** for fall prevention practice in older adults [14], participants will be provided with a tailored home-exercise program developed by a physiotherapist to gain an additional 1-hour of physical activity (e.g. 3 sessions of 20 min). Home-based exercises will be re-evaluated on a monthly basis by the study physiotherapist to ensure exercises are at the appropriate level of challenge. **Intervention fidelity (standardizing the delivery):** Instructors will undergo training on topics specific to working with older adults living with frailty.

Arm 3: Multi-modal Intervention:

Group Exercise/Supplemental Home Exercise: This will be delivered identically to Arm 2. **Oral Nutritional Supplementation:** As piloted in our Virtual Frailty Rehab Study, the ONS (each serving) contains 225kcal, and 11-12 grams protein (Glucerna, Ensure). Abbott Nutrition has provided support to this study in relation to the Abbott products Ensure® and Glucerna®. Participants are advised to take this with a meal or within 3 hours of exercise on activity days. Participants will be provided with two ONS each day of the study. The research staff will schedule Arm 3 participants for an initial and post assessment appointment. Working in consultation with the study geriatricians and pharmacist, as required, the multi-modal arm assessment will include: 1) Reviewing the participants' relevant medical and medication history at the baseline assessment (using the medical and medication history form completed and collected by study personnel). **Nutrition review**: Conduct nutritional screening flow (developed by a consultant dietician) and review any questions, provide some additional counseling/coaching at follow-up visits. ONS will be provided to all participants unless contraindicated. **Medication review:** Review/update current medication list (baseline medical history form) and forward the list and medical history to the consultant study pharmacist. Prior to the medication review, we will also be requesting permission from the participant to contact their family physician and community pharmacist to receive their medication list. The Beers and START/STOPP criteria will be considered to provide

recommendations, via letters, to the participant's family physician and community pharmacist. Research staff will follow up with participants regarding their medication lists to determine whether the medication recommendations were implemented.

All Arms:

There is good evidence that indicates that **vitamin D3** supplementation preserves muscle strength and functional ability in high-risk groups such as older adults with frailty and reduces the risk of falls [43,44]. Given that it is a low-cost, logistically easy intervention recommended in the management of frail patients, we have elected to give all arms this treatment (1000 IU/daily) for both ethical (a proven intervention in vulnerable cohorts) and practical reasons (many individuals already taking Vitamin D). Individuals already taking Vitamin D (1000 IU/or greater) will continue with prescribed treatments.

After participants' involvement in the study, all participants will receive complimentary YMCA memberships for a limited time. Participants, if they wish to, will also be offered free resources and an educational session.

Figure 1: Study Schema

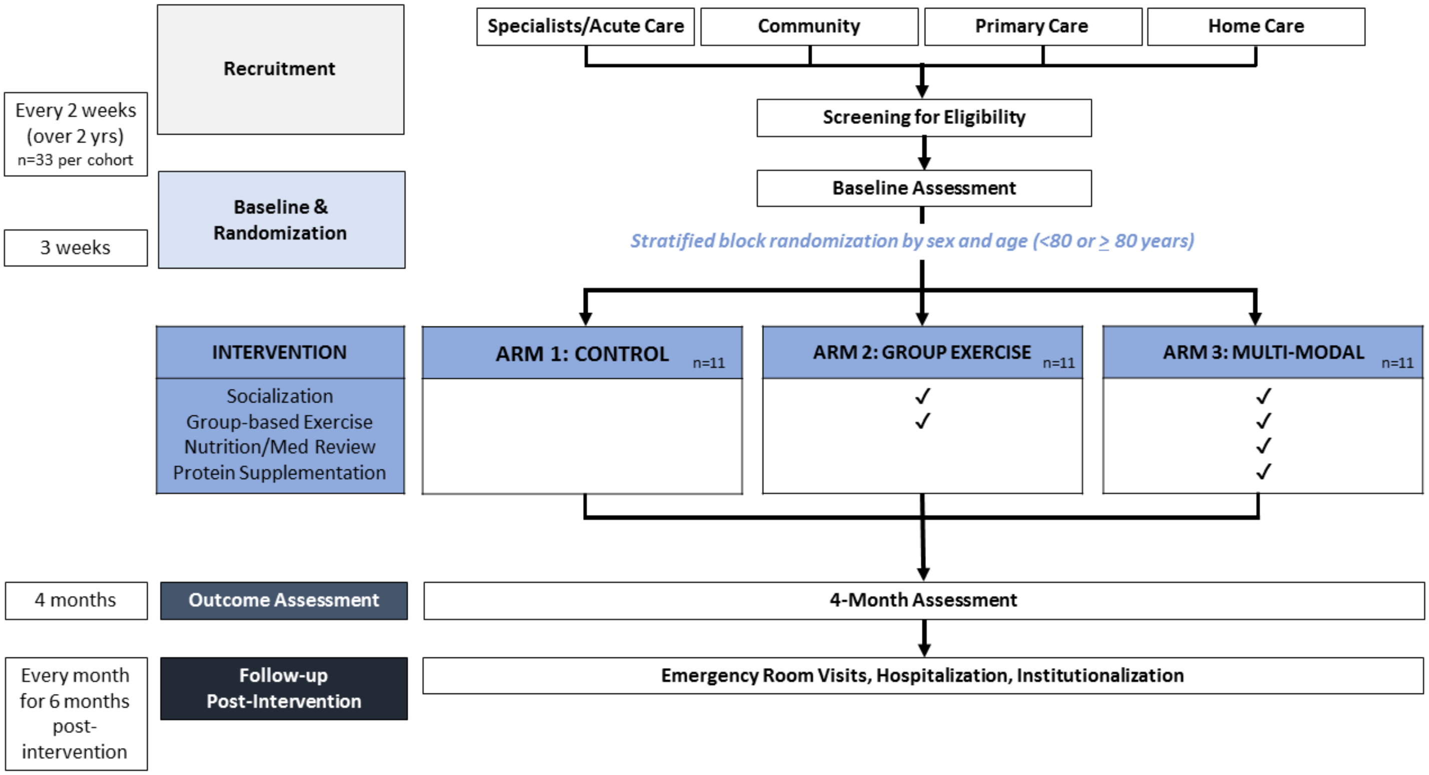

4.7. Monitoring Compliance: The study team is well aware of the barriers encountered with compliance when working with vulnerable older adults, which includes transportation challenges, caregiver burden, medical concerns, indifference, cognitive limitations and poor health [45]. Class attendance will be tracked via YMCA attendance monitoring. Participants will be asked to track weekly adherence to home and group exercises, nutrition (ONS, monthly diet food log, and vitamin D), and falls/adverse events in log-books. Study personnel will conduct monthly check-ins by phone to check-in on adverse events and to assess and assist with challenges pertaining to their intervention. In the GERAS DANCE pilot study for cognitively impaired/pre-frail older adults, we have overcome a number of recruitment challenges and we can apply this learning. For example, YMCA trained volunteers will greet and assist participants to ensure safety on arrival. Furthermore, this group of investigators has extensive experience running such trials and can draw on existing infrastructure and past experiences [e.g., Health TAPESTRY [46]; Cardiovascular Health Awareness Program [47]; Vitamin D and Osteoporosis Study (ViDOS) [48,49]].

4.8. Trial outcomes: Primary, secondary, and exploratory outcomes will be assessed at **0 and 4-months**. Healthcare utilization will be tracked (via monthly phone-call by research personnel) during the intervention phase and for an **additional 6-months** following the intervention. The primary outcome measures are the Short Performance Physical Battery (SPPB) [50] and 400m Walk Test [51] (**Table 2**). The **SPPB was the primary outcome of the first frailty intervention trial** [8] and demonstrated improvement in both the FIT study and the LIFE trial, the largest physical activity study in older adults, to date [52,53]. SPPB total scores range from 0-12, and ≤7 is an established cut-off for poor physical performance. A substantial clinical meaningful change in total SPPB score is 1.3 points [54]. A clinically significant change is considered in the range of 0.05 with 0.10 considered a substantial shift in scores [55].

A blinded assessor with kinesiology/rehabilitation training (with assistance from a GERAS volunteer who will be trained on safety supervision) will perform study outcome assessments at Hamilton Health Science (piloted in our DANCE study). Data, with identifying information removed, will be securely stored in a locked cabinet in the locked research office, at the GERAS Centre at Hamilton Health Sciences. Electronic data will be entered and stored in a **REDCap (Research Electronic Data Capture) database**.

A subset of participants, from all 3 study arms, will be randomly selected to wear the study ActiGraph GT9X Link Accelerometer for the duration of the study. The device will collect raw acceleration data, physical activity intensity, total sleep time, activity bouts, sedentary bouts, sleep efficiency, and energy expenditure, in real time. **Sarcopenia outcomes:** All participants will be screened for sarcopenia at baseline. Physical performance based on the 5-time chair stand test with a score of greater than or equal to 12 seconds is predictive of sarcopenia [56]. Of the 324 participants, up to 75 of them with sarcopenia will undergo additional assessments. Aligned with the 2019 Asian Working Group for Sarcopenia Consensus on Sarcopenia and Treatment we will assess, confirm and grade the severity of sarcopenia at baseline and 4-months. Muscle mass will be assessed with readily available dual-energy x-ray absorptiometry (DXA), and the gold standard magnetic resonance imaging (MRI). Up to 75 participants with sarcopenia will be assessed with DXA (**Table 2**). Due to budget constraints, a subset of up to 36 participants will undergo an MRI scan in addition to DXA. Muscle strength will be assessed as grip strength and chair stand tests. Muscle function will be quantified by gait speed, short physical performance

battery, timed-up and go, and 400m walk tests. Once the study has reached capacity of participants with sarcopenia, requisitions for DXA and MRI will no longer be provided.

Table 2: Primary and Secondary Outcomes Measures [See timeline in Appendix B]

**Primary**	**Physical Function**<br>[Baseline and 4-months]	Short Physical Performance Battery (SPPB)<br>400-m Walk Test [walking speed, m/s]
**Secondary**	**Functional Abilities**<br>[Baseline and 4-months]	Frailty: Fit-Frailty App<br>Fear of Falling: Icon Falls Efficacy Scale (ICON-FES)<br>Fitness: Modified Rating of Perceived Exertion (RPE) scale (post-400m walk test)<br>Strength: Grip strength with handgrip dynamometer [kg]<br>Cognition: Montreal Cognitive Assessment (MoCA)<br>Health-related quality of life: EQ-5D-5L<br>Life Space Mobility: Life Space Assessment<br>Activities of Daily Living: Katz ADL and Lawton IADL<br>Activity and Sleep Patterns: ActiGraph GT9X Link Accelerometer Data<br>Depression/Mood: Geriatric Depression Scale (GDS-SF)<br>Nutrition: Mini Nutritional Assessment and SCREEN-8<br>Functional Mobility: Timed Up and Go (TUG) Test
	**Sarcopenia**	Sarcopenia screening: 5-Time Chair Stand Test (SPPB)[Baseline and 4-month]<br>Sarcopenia: SARC-F [Baseline and 4-month]<br>Muscle Mass: DXA† and MRI* [Baseline and 4-month]<br>†Additional outcome assessed in up to 75 individuals with sarcopenia<br>*Additional outcome assessed in up to 36 individuals with sarcopenia
**Exploratory**	**Healthcare utilization**<br>[Baseline and 4-months and additional 6-month follow-up]	Number of emergency room visits<br>Number of hospitalizations & length of stay<br>Institutionalization (to long-term care)<br>*(measured during intervention phase and 6-months)*

4.9. Participant Feedback: We will provide all participants with a participation satisfaction survey designed to facilitate a conversation about individual motivators and barriers to participation and attitudes and behaviours surrounding the frailty rehabilitation program. Participant characteristics that will be collected include age, sex, gender [measured by the Physical Self-Attribute Questionnaire [57], chronic conditions, medication use (medication # [total, started, stopped], 25(OH)D vitamin D level (mmol/L), participation in other programs and sociodemographic descriptors. Adherence will be examined in sub-analyses.

4.10 Study Staff Process Evaluation: We will be interviewing at least 4 study staff designed to assist in the evaluation of the intervention processes. The interview will be approximately 30 minutes long.

5.0. SAMPLE SIZE

In the FIT trial [15], the mean baseline score was approximately 5.5 on the SPPB, however, participants were

recently discharged from inpatient units. In our geriatric outpatient clinic study, the mean baseline score was 6.3. Assuming baseline scores of approximately 6, we aim to improve or maintain group exercise and socialization scores (and not decline over time as it did in the FIT Trial control group) and achieve at least a 1.5 between-group difference with the multi-modal arm. **Improving SPPB scores by 1.3-points is substantial clinical meaningful change** [54] **and bringing scores above 7 represents an important functional difference** [InCHIANTI study, scores below 7 were strongly associated with mobility disability which limits independence in the community [58]]. **<u>Sample size (SPPB):</u>** Assuming at least a 1-point difference at 6-month follow-up (e.g. SPPB= 6.5 in group exercise versus 7.5 in multi-modal, SD=2 based on FIT trial and our own pilot data), with alpha 0.05 and power of 90%, and factoring in a drop-out rate of 20%, we will need a total sample size of N=324 (108 per arm). **<u>Sample size (Frailty):</u>** Assuming at least a 0.05-point difference at 6-month follow-up (e.g. Frailty score 0.25 in group exercise versus 0.20 in multi-modal, SD=0.09 based on our CaMos study data [3], with alpha 0.05 and power of 90%, and factoring in a drop-out rate of 20%, we will need a total sample N=195 (65 per arm). **To achieve the power to examine both outcomes, we will target a total cohort of 324.**

In addition to examining important scientific questions related to the effectiveness of frailty rehabilitation and functional capabilities of older adults living with frailty, this study will examine whether frailty rehabilitation is a sustainable community-based model that decreases healthcare utilization. Significant societal benefits could be achieved if hospitalization and institutionalization can be postponed or avoided. Furthermore, with limited access to geriatric services, examining other models for supporting primary care providers in managing frailty patients are imperative.

6.0. ANALYSIS PLAN

The study statistician will analyze the study data and report it in accordance with the CONSORT criteria after data collection is completed. The analysis plan is described in **Table 3**. The **primary comparisons** are at 4-months. We will report between-group differences in, means or percentages, with 95% confidence intervals at the 4-month follow-up. All analysis will be an intention to treat. ***Sensitivity Analyses:*** 1) we will examine the per-protocol cohort (participants who completed the trial). 2) To examine the influence of covariates on our dependent variables including adherence, the generalized estimating equations (GEE) technique, assuming an autoregressive correlation structure, will be used to compare differences between intervention groups. GEE is a flexible tool to deal with correlated data (across multiple time-points) and appropriate for both continuous and dichotomous outcomes.

Table 3: Analysis Plan of Co-Primary Outcomes

Objectives	Hypotheses	Outcomes	Analysis
In community-dwelling older adults with frailty, does frailty rehabilitation (multi-modal and exercise only), compared with control (daily activity), **improve physical function measured by physical performance**?	Improved physical function with intervention; greatest effect in the multi-modal	SPPB (total score, sub-component scores) at 4-months **Sub-group:** Comparison b/w intervention groups; adherence	one-way ANOVA, post-hoc analysis GEE
In community-dwelling older adults with frailty, does frailty rehabilitation (multi-modal and group exercise only), compared with control (daily activity), **improve physical function measured by walking speed**?	Improved frailty status with intervention; greatest effect in the multi-modal	400m Walk Test (continuous) scores; **Sub-group:** Comparison b/w intervention groups; adherence	one-way ANOVA, post-hoc analysis GEE

Economic analysis: An individual-level economic evaluation alongside this trial will be done from the Ministry of Health perspective. We will examine changes in direct medical costs and effectiveness outcomes (e.g., quality-adjusted life years - QALYs), calculate and compare the ICER (e.g., $/ QALY gained, $/ a visit averted)[59] against a willingness-to-pay threshold ($50,000/QALY)[60] to show if this program of frailty rehabilitation represents good value for money.

7.0. TRIAL MANAGEMENT

The coordinating centre for the study is at the GERAS Centre for Aging Research at Hamilton Health Sciences. Overseen by the scientists and post-doctoral candidate, the research staff will be responsible for initiating the randomization procedure, submitting/maintaining REB documents, scheduling assessments, check-in phone-calls, receiving and storing consent forms. The study Steering Committee will meet every 6 months to provide overall supervision of the trial. The research assistant will call more frequent Steering Committee meetings if required. The **steering committee** will consist of: Dr. **Marr,** Director of Geriatric Medicine at McMaster with extensive experience in inter-professional education. Dr. **Tarride**, a health economist and will oversee economic analysis. Dr. **Rockwood**, a geriatrician and international leader in frailty and dementia. Dr. **Theou** is a Gerokinesiologist whose research is focused on aging, frailty, and their interaction with physical activity. Dr. **Mangin** is a Family Physician and Director of Research for Family Medicine at McMaster and is the lead author of TAPER (TaperMD.com). Dr. **Dolovich** is a pharmacist and one of the founding leads of the 8.7 million dollar Health TAPESTRY primary care initiative. Dr. **Adachi** is an international leader in osteoporosis, osteoarthritis, and sarcopenia. Dr. **Thabane** is a biostatistician and international expert in trial methodology. Dr. **Kaasalainen** is a nurse researcher with expertise in qualitative analysis and public health/primary care collaboration. A **Data and Safety Monitoring Board (DSMB)** will oversee the study (bi-annual meetings), which can recommend changes to the protocol or termination of the study.

Upon request, the research staff will submit quarterly oral or written reports on study progress including but not limited to information on study subject enrollment, publication plans, and any adjustments in the estimated study completion date. Within 45 days following the completion or termination of the study, the

research staff will submit a final report detailing the results of the study to Abbott Nutrition and Abbott Nutrition representatives. The study reports shall be considered confidential information of McMaster University.

8.0. POTENTIAL RISK AND BENEFITS

Risks: The potential risks of the study are reasonable. There may be a slightly greater risk of falling, musculoskeletal injury and/or fatigue with engaging in exercise classes and assessments of physical function/frailty. To minimize risks, participants will be cleared for exercise by their primary care physician or referring physician prior to beginning; classes will be tailored to meet the varied needs of frail older adults and administered by expert exercise trainers and the study physiotherapist. Further, the use of walking poles in-class, apart from its efficacy, is also due to the safety and stability it offers. At any time if participants feel uncomfortable, they may decline participation at any time.

For participants that are selected and have consented to wear an ActiGraph accelerometer, there is a small risk of a privacy breach for data collected on the external servers. ActiGraph complies with the Health Insurance Portability and Accountability Act of 1996 ("HIPAA") and its amendments to ensure the protection of Protected Health Information ("PHI"). Safeguards that are currently used to ensure the protection of private health information include administrative procedures, physical data safeguards, electronic data access security, and network security that complies with legal requirements.

For participants who choose to provide an electronic signature via DocuSign, DocuSign is fully compliant with all data residency requirements and data privacy legislation governed federally under PIPEDA.

For potential participants who choose to self-refer to the Geras Centre website, the website has an AES-256 bank/military grade database storage encryption and has security measures in place to help prevent sensitive data breaches.

Benefits: If we can prove that frailty rehabilitation within our setting was effective, the next phase is to consider the generalizability of our approach, i.e., how this model could be applied within communities of varied size, demographic composition, geographical location. Our avoidance of specialized geriatric assessment teams allows for the potential for uptake in smaller centres and our partnership with the YMCA could help with pan-Canadian expansion. There are 120 Health & Fitness Centres across Canada serving 1.2 million Canadians of all ages. Further, the YMCA is a charitable organization that believes programs should be accessible to everyone regardless of age, background, ability or socio-economic status (e.g., fee assisted memberships are available). Our frailty rehabilitation model could be generalized to other community centres to increase the program accessibility.

9.0 ADVERSE EVENTS (AE)

Participants will be instructed to contact the study coordinator if they experience any unfavorable/unintended signs or symptoms. Any untoward medical occurrence in a research participant, administered investigational product, including an occurrence which does not have a causal relationship with the product. An AE can be any unfavorable and unintended sign, symptom, or disease temporally associated with the use of an investigational product, or not related to the investigational product. Adverse events or harm in this study such as falls, fracture,

pain with exercise, dizziness, chest pain, death or any other event from any source will be reported to the research team and recorded on a structured form. If applicable under the regulations governing the study, the research staff shall report adverse events that occur during the course of the study to Abbott Nutrition. The research staff shall also report all periodic reports regarding the study to Abbott Nutrition along with any safety issues related to the study or safety signal associated with the Abbott Nutrition study product.

9.1 SERIOUS ADVERSE EVENTS (SAE)

Any local serious adverse events that in the opinion of the local Principal Investigator is both unexpected and related or possibly related to, the study intervention or research procedures, must be reported to the REB.
If any adverse events meet the criteria for a serious adverse event such as a fatal, life threatening event resulting in significant or persistent disability/incapacity, requiring or prolonging hospitalization or presenting other significant hazards or potentially serious harm in the judgment of the site investigator will be reported to the Research Ethics Board within 7 business days of becoming aware of the event.

10.0. TRIAL REGISTRATION

The trial has been registered with ClinicalTrials.gov NCT03824106.

11.0. REFERENCES

1. Fried LP, Tangen CM, Walston J, Newman AB, Hirsch C, Gottdiener J, et al. Frailty in Older Adults: Evidence for a Phenotype. J Gerontol A Biol Sci Med Sci. 2001;56(3):M146–57.
2. Rockwood K, Mitnitski A. Frailty Defined by Deficit Accumulation and Geriatric Medicine Defined by Frailty. Vol. 27, Clinics in Geriatric Medicine. 2011. p. 17–26.
3. Kennedy C, Ioannidis G, Rockwood K, Thabane L, Adachi J, Kirkland S, et al. A Frailty Index predicts 10-year fracture risk in adults age 25 years and older: results from the Canadian Multicentre Osteoporosis Study (CaMos). Osteoporosis International. 2014;25(12):2825–32.
4. Rockwood K, Song X, Mitnitski A. Changes in relative fitness and frailty across the adult lifespan: Evidence from the Canadian National Population Health Survey. CMAJ. 2011;183(8).
5. Roberts KC, Rao DP, Bennett TL, Loukine L, Jayaraman GC. Prevalence and patterns of chronic disease multimorbidity and associated determinants in Canada. Chronic Dis Inj Can. 2015;35(6):87–94.
6. Rockwood K, Howlett SE, MacKnight C, Beattie BL, Bergman H, Hébert R, et al. Prevalence, attributes, and outcomes of fitness and frailty in community-dwelling older adults: report from the Canadian study of health and aging. J Gerontol A Biol Sci Med Sci. 2004;59(12):1310–7.
7. Rockwood K, Song X, Mitnitski A. Changes in relative fitness and frailty across the adult lifespan: Evidence from the Canadian National Population Health Survey. CMAJ. 2011;183(8).
8. Cameron ID, Fairhall N, Langron C, Lockwood K, Monaghan N, Aggar C, et al. A multifactorial interdisciplinary intervention reduces frailty in older people: randomized trial. BMC Med. 2013;11(1):65.
9. Morley JE, Vellas B, Abellan van Kan G, Anker SD, Bauer JM, Bernabei R, et al. Frailty conensus: a call to action. Journal of the American Medical Directors Assocation. 2014;14(6):392–7.
10. Anderson L, Oldridge N, Thompson DR, Zwisler A-D, Rees K, Martin N, et al. Exercise-based cardiac rehabilitation for coronary heart disease: Cochrane systematic review and meta-analysis. J Am Coll Cardiol. 2016;67(1):1–12.
11. Dalal HM, Doherty P, Taylor RS. Cardiac rehabilitation. BMJ. 2015;1–8.
12. Cheifetz O, Park Dorsay J, Hladysh G, MacDermid J, Serediuk F, Woodhouse LJ. CanWell: Meeting the psychosocial and exercise needs of cancer survivors by translating evidence into practice. Psychooncology. 2014;23(2):204–15.
13. Cheifetz O, Dorsay JP, MacDermid JC. Exercise facilitators and barriers following participation in a community-based exercise and education program for cancer survivors. J Exerc Rehabil. 2015;11(1):20–9.
14. Sherrington C, Michaleff ZA, Fairhall N, Paul SS, Tiedemann A, Whitney J, et al. Exercise to prevent falls in older adults: an updated systematic review and meta-analysis. Br J Sports Med. 2016;bjsports-2016-096547.
15. Fairhall N, Aggar C, Kurrle SE, Sherrington C, Lord S, Lockwood K, et al. Frailty Intervention Trial (FIT). BMC Geriatr. 2008;8(1):27.

16. Ng TP, Feng L, Nyunt MSZ, Feng L, Niti M, Tan BY, et al. Nutritional, Physical, Cognitive, and Combination Interventions and Frailty Reversal among Older Adults: A Randomized Controlled Trial. American Journal of Medicine. Elsevier Inc; 2015;128(11):1225–36.

17. Kelaiditi E, Cesari M, Canevelli M, Abellan Van Kan G, Ousset PJ, Gillette-Guyonnet S, et al. Cogntive frailty: Rationale and Definition fom an (I.A.N.A./I.A.G.G.) international consensus group. Journal of Nutrition, Health & Aging. 1991;17(9):262.

18. World Health Organization (WHO). Summary: World Report on Aging and Health [Internet]. 2015.

19. World Health Organization (WHO). Summary: World Report on Aging and Health [Internet]. 2015.

20. Cesari M. Intersections between Frailty and the Concept of Intrinsic Capacity. Innov Aging. 2017;1:S1.

21. Brach M, Moschny A, Bücker B, Klaaßen-Mielke R, Trampisch M, Wilm S, et al. Recruiting hard-to-reach subjects for exercise interventions: A multi-centre and multi-stage approach targeting general practitioners and their community-dwelling and mobility-limited patients. Int J Environ Res Public Health. 2013;10(12):6611–29.

22. Burns A, Brayne C, Folstein M. Mini-Mental State: A practical method for grading the cognitive state of patients for the clinician. M. Folstein, S. Folstein and P. McHugh, Journal of Psychiatric Research (1975) 12, 189-198. Vol. 13, International Journal of Geriatric Psychiatry. 1998. p. 285–94.

23. Negm AM, Kennedy CC, Thabane L, Veroniki A-A, Adachi JD, Richardson J, et al. Management of frailty: a protocol of a network meta-analysis of randomized controlled trials. Syst Rev. Systematic Reviews; 2017;6(1):130.

24. Novek S, Menec V, Tran T BS. Exploring the Impacts of Senior Centres on Older Adults. Winnepeg, MB;

25. Haslam C, Cruwys T, Haslam SA. “The we’s have it”: Evidence for the distinctive benefits of group engagement in enhancing cognitive health in aging. Soc Sci Med. 2014;120:57–66.

26. Papaioannou A, Kennedy CC, Giangregorio L, Ioannidis G, Pritchard J, Hanley DA, et al. A randomized controlled trial of vitamin D dosing strategies after acute hip fracture: No advantage of loading doses over daily supplementation. BMC Musculoskelet Disord. 2011;12.

27. Visser M, Deeg DJH, Lips P. Low Vitamin D and High Parathyroid Hormone Levels as Determinants of Loss of Muscle Strength and Muscle Mass (Sarcopenia): The Longitudinal Aging Study Amsterdam. Journal of Clinical Endocrinology and Metabolism. 2003;88(12):5766–72.

28. Houston DK, Cesari M, Ferrucci L, Cherubini A, Maggio D, Bartali B, et al. Association between vitamin D status and physical performance: the InCHIANTI study. J Gerontol A Biol Sci Med Sci. 2007;62(4):440–6.

29. Bruyère O, Cavalier E, Souberbielle J-C, Bischoff-Ferrari H a, Beaudart C, Buckinx F, et al. Effects of vitamin D in the elderly population: current status and perspectives. Arch Public Health. 2014;72(1):32.

30. Muir SW, Montero-Odasso M. Effect of vitamin D supplementation on muscle strength, gait and balance in older adults: A systematic review and meta-analysis. J Am Geriatr Soc. 2011;59(12):2291–300.

31. Stockton KA, Mengersen K, Paratz JD, Kandiah D, Bennell KL. Effect of vitamin D supplementation on muscle strength: a systematic review and meta-analysis. Osteoporos Int. 2011;22(3):859–71.

32. Halfon M, Phan O, Theta D. Vitamin D: A review on its effects on muscle strength, the risk of fall, and frailty. Biomed Res Int. 2015;2015.

33. Cadore EL, Rodríguez-Mañas L, Sinclair A, Izquierdo M. Effects of Different Exercise Interventions on Risk of Falls, Gait Ability, and Balance in Physically Frail Older Adults: A Systematic Review. Rejuvenation Res. 2013;16(2):105–14.

34. Sherrington C, Michaleff ZA, Fairhall N, Paul SS, Tiedemann A, Whitney J, et al. Exercise to prevent falls in older adults: an updated systematic review and meta-analysis. Br J Sports Med. 2016;bjsports-2016-096547.

35. Kramer IF, Verdijk LB, Hamer HM, Verlaan S, Luiking YC, Kouw IWK, et al. Both basal and post-prandial muscle protein synthesis rates, following the ingestion of a leucine-enriched whey protein supplement, are not impaired in sarcopenic older males. Clinical Nutrition. 2016;

36. Drey M, Pfeifer K, Sieber CC, Bauer JM. The fried frailty criteria as inclusion criteria for a randomized controlled trial: Personal experience and literature review. Gerontology. 2010;57(1):11–8.

37. Tieland M, van de Rest O, Dirks ML, van der Zwaluw N, Mensink M, van Loon LJC, et al. Protein Supplementation Improves Physical Performance in Frail Elderly People: A Randomized, Double-Blind, Placebo-Controlled Trial. J Am Med Dir Assoc. 2012;13(8):720–6.

38. Kim C-O, Lee K-R. Preventive effect of protein-energy supplementation on the functional decline of frail older adults with low socioeconomic status: a community-based randomized controlled study. J Gerontol A Biol Sci Med Sci. 2013;68(3):309–16.

39. Tieland M, van de Rest O, Dirks ML, van der Zwaluw N, Mensink M, van Loon LJC, et al. Protein Supplementation Improves Physical Performance in Frail Elderly People: A Randomized, Double-Blind, Placebo-Controlled Trial. J Am Med Dir Assoc. 2012;13(8):720–6.

40. Volkert D, Beck AM, Cederholm T, Cruz-Jentoft A, Hooper L, Kiesswetter E, et al. ESPEN practical guideline: Clinical nutrition and hydration in geriatrics. Clin Nutr. 2022 Apr;41(4):958–89.

41. Cooper JA, Cadogan CA, Patterson SM, Kerse N, Bradley MC, Ryan C, et al. Interventions to improve the appropriate use of polypharmacy in older people: a Cochrane systematic review. BMJ Open. 2015;5(12):e009235.

42. Page AT, Clifford RM, Potter K, Schwartz D, Etherton-Beer CD. The feasibility and effect of deprescribing in older adults on mortality and health: a systematic review and meta-analysis. British Journal of Clinical Pharmacology. 2016. p. 583–623.

43. Bischoff-Ferrari HA, Dawson-Hughes B, Staehelin HB, Orav JE, Stuck AE, Theiler R, et al. Fall prevention with supplemental and active forms of vitamin D: a meta-analysis of randomised controlled trials. BMJ. 2009;339(oct01 1):b3692–b3692.

44. Muir SW, Montero-Odasso M. Effect of vitamin D supplementation on muscle strength, gait and balance in older adults: A systematic review and meta-analysis. J Am Geriatr Soc. 2011;59(12):2291–300.

45. McHenry JC, Insel KC, Einstein GO, Vidrine AN, Koerner KM, Morrow DG. Recruitment of older adults: Success may be in the details. Gerontologist. 2015;55(5):845–53.

46. Dolovich L, Oliver D, Lamarche L, Agarwal G, Carr T, Chan D, Cleghorn L, Griffith L, Javadi D, Kastner M, Longaphy J, Mangin D, Papaioannou A, Ploeg J, Raina P, Richardson J, Risdon C, Santaguida PL, Straus S, Thabane L, Valaitis R, Price D. A protocol for a pragmatic randomized controlled trial using the Health Teams Advancing Patient Experience: Strengthening Quality (Health TAPESTRY) platform approach to promote person-focused primary healthcare for older adults. Implement Sci. 2016 Apr 5;11:49.

47. Kaczorowski J, Chambers LW, Karwalajtys T, Dolovich L, Farrell B, McDonough B, et al. Cardiovascular Health Awareness Program (CHAP): A community cluster-randomised trial among elderly Canadians. Prev Med (Baltim). 2008;46(6):537–44.

48. Kennedy CC, Thabane L, Ioannidis G, Adachi JD, Papaioannou A. Implementing a Knowledge Translation Intervention in Long-Term Care: Feasibility Results From the Vitamin D and Osteoporosis Study (ViDOS). J Am Med Dir Assoc. 2014;15(12):943–5.

49. Kennedy CC, Ioannidis G, Giangregorio LM, Adachi JD, Thabane L, Morin SN, et al. An interdisciplinary knowledge translation intervention in long-term care: Study protocol for the vitamin D and osteoporosis study (ViDOS) pilot cluster randomized controlled trial. Implementation Science. 2012;7(1):48.

50. Guralnik JM, Simonsick EM, Ferrucci L, Glynn RJ, Berkman LF, Blazer DG, et al. A short physical performance battery assessing lower extremity function: association with self-reported disability and prediction of mortality and nursing home admission. J Gerontol. 1994;49(2):85–94.

51. Vestergaard S, Patel K V., Bandinelli S, Ferrucci L, Guralnik JM. Characteristics of 400-Meter Walk Test Performance and Subsequent Mortality in Older Adults. Rejuvenation Res. 2009;12(3):177–84.

52. Pahor M, Blair SN, Espeland M, Fielding R, Gill TM, Guralnik JM, et al. Effects of a physical activity intervention on measures of physical performance: Results of the lifestyle interventions and independence for Elders Pilot (LIFE-P) study. J Gerontol A Biol Sci Med Sci. 2006;61(11):1157–65.

53. Fielding RA, Rejeski WJ, Blair S, Church T, Espeland MA, Gill TM, et al. The lifestyle interventions and independence for elders study: Design and methods. Journals of Gerontology - Series A Biological Sciences and Medical Sciences. 2011;66 A(11):1226–37.

54. Kwon S, Perera S, Pahor M, Katula JA, King AC. Meaningfulness in Performance Measures What Is a Meaningful Change in Physical Performance ? Findings From a Clinical Trial in Older Adults (the Life-P Study). 2009;13(6):538–45.

55. Kennedy CC, Ioannidis G, Rockwood K, Thabane L, Adachi JD, Kirkland S, et al. A Frailty Index predicts 10-year fracture risk in adults age 25 years and older: results from the Canadian Multicentre Osteoporosis Study (CaMos). Osteoporosis International. 2014;25(12):2825–32.

56. Chen LK, Woo J, Assantachai P, Auyeung TW, Chou MY, Iijima K, Jang HC, Kang L, Kim M, Kim S, Kojima T, Kuzuya M, Lee JSW, Lee SY, Lee WJ, Lee Y, Liang CK, Lim JY, Lim WS, Peng LN, Sugimoto K, Tanaka T, Won CW, Yamada M, Zhang T, Akishita M, Arai H. Asian Working Group for Sarcopenia: 2019 Consensus Update on Sarcopenia Diagnosis and Treatment. J Am Med Dir Assoc. 2020 Mar;21(3):300-307.

57. Moore J, Mitchell N, Kilpatrick M, Bartholomew J. The Physical Self-Attribute Questionnaire: Development and Inital Validation. Psychol Rep. 2007;100:627–42.

58. Vasunilashorn S, Coppin AK, Patel K V., Lauretani F, Ferrucci L, Bandinelli S, et al. Use of the short physical performance battery score to predict loss of ability to walk 400 meters: Analysis from the InCHIANTI study. Journals of Gerontology - Series A Biological Sciences and Medical Sciences. 2009;64(2):223–9.

59. Drummond M, Sculpher MJ, Torrance GW, BJ O, Stoddart GL. Methods for the economic evaluation of healthcare programmes. Methods for the Economic Evaluation of Health Care Programmes. 2015. 379 p.

60. Gafni A, Birch S. Incremental cost-effectiveness ratios (ICERs): The silence of the lambda. Soc Sci Med. 2006;62(9):2091–100.

Appendix A: Clinician Pre-Screening/Study Referral Form

OPTIMAL Fitness
Optimizing Independence, Mobility and Active Life

CLINICIAN PRE-SCREENING / STUDY REFERRAL FORM
Page 1 of 2, July 11, 2022, V2.5

Completed by:_______________________
Date: ___________________________
Unit: ___________________________

Study Description: Older adults often have low muscle strength and/or muscle loss (sarcopenia), slower walking speed, recent weight loss and/or low levels of physical activity. Studies have shown that a person's level of physical fitness can be improved in response to exercise only or multimodal interventions including exercise. Our aim is to consider if a rehabilitation program is feasible and effective for older adults living with frailty who are community-dwelling. More specifically the purpose of this study is to learn if a multi-modal rehabilitation program improves physical function and reduces frailty and/or sarcopenia in community-dwelling older adults compared to usual care and exercise alone.

1) PLEASE COMPLETE THE FOLLOWING PRE-SCREENING CRITERIA FOR THE PATIENT:

Inclusion Criteria (must answer YES to be eligible)		Exclusion Criteria (must answer NO to be eligible)	
☐ YES to ALL Inclusion Criteria		☐ NO to ALL Exclusion Criteria	
1) Lives in the community (not LTC)	☐NO ☐ YES	1) Currently attending a group exercise program	☐NO ☐ YES
2) ≥65 years of age	☐NO ☐ YES	2) Unable to speak or understand English	☐NO ☐ YES
3) Medical clearance from referring clinician to safely participate in exercise	☐NO ☐ YES	3) Significant cognitive impairment, unable to follow 2-step commands	☐NO ☐ YES
4) Medical clearance from referring clinician to safely take oral nutritional supplements	☐NO ☐ YES	4) Currently in a drug optimization study/program	☐NO ☐ YES
5) Able to ambulate 25m with or without a walking aid by study screening visit	☐NO ☐ YES	5) Currently taking oral nutritional supplements daily	☐NO ☐ YES
6) Can arrange transportation to YMCA up to 2x/week	☐NO ☐ YES	6) Diagnosis of unstable angina or heart failure	☐NO ☐ YES
		7) Receiving palliative/end of life care	☐NO ☐ YES

OPTIMAL Fitness — Optimizing Independence, Mobility and Active Life

CLINICIAN PRE-SCREENING / STUDY REFERRAL FORM
Page 2 of 2, July 11, 2022, V2.5

Completed by:______________________
Date: ________________________
Unit: ________________________

7) At risk for mobility/functional limitations as assessed by the John Morley FRAIL Scale. Has **2 or more** of the following markers of frailty: ☐ NO ☐ YES<br>1. Difficulty climbing stairs, slow gait speed (e.g. have begun walking slower, or difficulty walking several hundred yards or about 2 kilometers)<br>2. Recent weight loss (unintentional)<br>3. Declines in energy (feel tired or fatigued often) and/or low physical activity<br>4. Previously diagnosed with 5 or more of the following illnesses: hypertension, diabetes, cancer, chronic lung disease, heart attack, congestive heart failure, angina, asthma, arthritis, stroke, kidney disease<br>**Frailty will be formally assessed at the study-screening visit*	8) Terminally ill ☐ NO ☐ YES<br>9) Another household member enrolled in the study ☐ NO ☐ YES<br>10) Unable to attend for more than 20% of the trial duration ☐ NO ☐ YES

Patient consent for information release and for study team to contact patient:

1. Patient gives consent for their name, contact information (phone and email), and the medical information above to be sent to the OPTIMAL Fitness study team.

2. Patient gives their verbal consent for a member of the study team to contact them about participating in the OPTIMAL Fitness study.

☐ **YES**

PLACE PATIENT ID STICKER HERE

OR PATIENT CONTACT INFORMATION:
(*NAME(S)/PHONE NUMBER(S)*)

____

Once complete, please **fax this form** to the confidential study fax machine at GERAS Centre:
905-318-2654

HAHSO — Hamilton Academic Health Sciences Organization

CIHR IRSC — Canadian Institutes of Health Research / Instituts de recherche en santé du Canada

If you have any questions, please phone the GERAS Frailty Rehab Research Staff at: 905-521-2100 ext. 12437

Appendix B: Study Assessments and Timepoints

		**STUDY PERIOD**		
	**Enrolment and Allocation**		**Post-allocation**	
**TIMEPOINT**	Screening,	Baseline Assessment and Randomization	4-months Assessment	6-month Assessment (telephone follow-up)
**Visit Window**	N/A	+/- 2-3 weeks	+/- 2-3 weeks	+/- 2-3 weeks
**ENROLMENT:**				
Informed Consent	X			
Eligibility screen	X			
Contact Form	X			
Randomization		X		
**ASSESSMENTS:**				
Demographics[1]		X		
Healthcare Use[2] and Medications and Supplements		X	X	X
Physical Measurements[3]		X	X	
Physical Function[4] and Muscle Strength[5]		X	X	
Frailty[6]		X	X	
Mobility[7] and Sarcopenia[8]		X	XX	
Fear of Falling[9]		X	X	
Barriers to Exercise and Transportation[10]		X		
Lab Blood Work		X	X	

Cognition[11]		X	XX	
QoL[12] & ADL/IADLs[13]		X	X	
Life Space Mobility[14]		X	XX	
Depression/ Mood[15]		X	XX	
Nutrition[16]		X	X	
Functional Mobility[17]		X	X	
Accelerometer Data		X	X	
Muscle Mass[18]		X [†]	X [†]	
Falls and Adverse events (monthly)			XX	X

[1]Demographics= Age, Gender, Housing, Education, Smoking Status, Falls, Hospitalizations, Walking Aid, Fractures, Past Surgeries, Chronic Conditions, Dietary Supplements, Multivitamins, Calcium supplements, Oral Nutritional Supplements, Physical Activity.
[2]Healthcare Use= number of GP visits/ walking clinic, number of specialist visits, number of physiotherapy visits
[3]Physical Measurements= height and weight, heart rate and blood pressure
[4]Physical Function= Short Physical Performance Battery (SPPB)
[5]Muscle Strength = grip strength with handgrip dynamometer [kg]
[6]Frailty Measures= Frailty Index (as measured by the GERAS Fit-Frailty App)
[7]Mobility= 400m walk test and Modified Rating of Perceived Exertion (RPE) Scale post-400m walk test.
[8]Sarcopenia= SARC-F questionnaire, 5-time chair stand test (via SPPB)
[9]Fear of Falling= Icon Falls Efficacy Scale (ICON-FES)
[10]Barriers to exercise and transportation= interest in having a healthy level of activity and eating well, exercise barriers, side effects to exercise, facilitators to exercise, barriers to transportation to the YMCA
[11]Cognition= Montreal Cognitive Assessment (MoCA)
[12]QoL (Quality of Life)= EQ-5D-5L Questionnaire
[13]ADL/IADLs= Katz Activities of Daily Living (ADL) and Lawton Instrumental Activities of Daily Living (IADLs)
[14]Life Space Mobility= Life Space Questionnaire
[15]Depression/ Mood= Geriatric Depression Scale (GDS-SF)
[16]Nutrition=Mini Nutritional Assessment (MNA) and SCREEN-8
[17]Functional Mobility= Timed Up and Go (TUG) Test
[18]Muscle Mass = Dual X-Ray Absorptiometry (DXA) and Magnetic Resonance Imaging (MRI)

[†] Up to 75 participants screened with sarcopenia will undergo DXA and up to 36 participants of those 75 will undergo an MRI at a time separate from the primary baseline assessments.

Appendix C: MIRA Voice Recruitment

This section outlines a web-based self-referral through advertising recruitment pathway for this study:

This project will also be advertised on the Voice platform. Voice is a community engagement platform that the McMaster Institute for Research on Aging (MIRA) is licensing from the University of Newcastle. The platform acts as a place where researchers can work with community members, through community engagement opportunities and features built-into the website, and though posted research studies, to collect insights and ideas that can be mobilized to drive innovation in research on aging forward. The platform includes an area specifically designed for researchers to post studies seeking participants. These postings are approved by staff at MIRA, who will verify that posted studies have been approved by relevant ethics bodies at the university. Users from the public register for Voice and provide both their contact information and personal information, including age, postal code, gender, sexuality, ethnicity, and employment status, when they do so. This information is stored in a database that can be accessed by staff at MIRA. Information from this database is stored on data servers based in the United Kingdom, hosted and controlled by the University of Newcastle. The privacy and data storage policies have been reviewed and approved by the privacy and legal offices of both McMaster University and the University of Newcastle. Marketing of the platform is primarily aimed to older adults (individuals over the age of 65), however anyone over the age of 18 can create an account. The Voice platform and was reviewed and approved by a joint MREB and HiREB committee as HiREB Application #14929.

Members of the public will find out about ads on Voice either through directly accessing the website, or through digest emails the platform sends out of new opportunities to registered users. After they click on an opportunity and review it, users must click to explicitly consent to sharing their name and email with researchers before they apply. Applicants are informed that researchers will follow up with them directly regarding their application. As part of the platform, researchers will receive the names and contact information of interested participants who apply to participate in opportunities.

Potential study participants will be contacted by one of the contact methods they provided (i.e., phone, email, and/or mailing address) within two weeks of Geras research staff receiving the notice of interest.

Voice has rigorous guidelines in place to both protect data and ensure there is a rapid response in the unlikely event of a security breach. To date, Voice has not experienced a security breach. Voice is well-supported by an expert IT and user design team based out of the University of Newcastle that prioritizes the security and protection of user data. Data Security Protocols were reviewed and approved as part of the platform's HiREB Application (#14929) and were also reviewed and approved by McMaster University's Privacy Officer and Legal Office before the platform's licensing agreement was signed.

Participant information obtained through the voice will be stored and dealt with in the same manner as all other study data. Please see the below image for more information on the Voice's consent process, and the attached document "Question Template for VOICE_OPTIMAL Study" that contains study information that will be posted on the Voice platform.

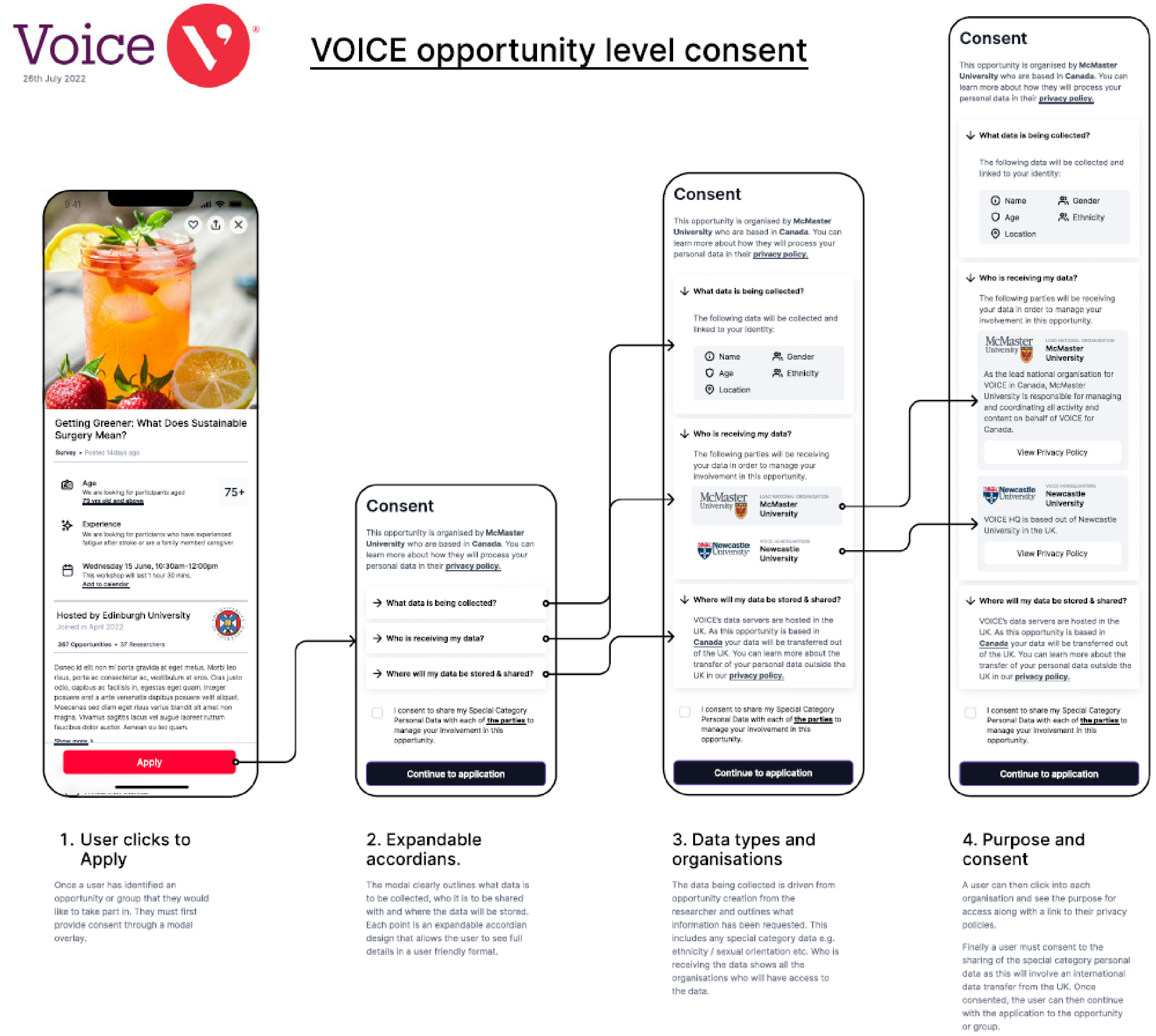

Larger Image.pdf
