# Supplementary material for: Study protocol for a multiarm, randomized controlled trial to determine the effectiveness of community-based frailty rehabilitation to improve physical function in older adults: The OPTIMAL Fitness Trial
Source: PLoS One. 2026 Mar 12;21(3):e0343338. doi: 10.1371/journal.pone.0343338 (PMC12981438; doi:10.1371/journal.pone.0343338)
Supplement: S3 File — (PDF) [file pone.0343338.s003.pdf]

**Frailty Rehab**	**Date Completed:**	**CRF-10**
**Participant ID #:**	**Completed By (Initials):**	
**Time-Point:** ☐ **Baseline** ☐ **Post-Intervention**		

Fit Frailty Index

Instructions:

Please indicate which visit you are completing the form for (i.e. baseline or post-intervention) at the top of the CRF.

(DEMOGRAPHIC/GENERAL HEALTH)		
1	Height	Record height<br>____________<br>☐ DK/refuse
2	Weight	Record weight<br>____________<br>☐ DK/refuse
Were height/weight measured at the time of the assessment?		☐ Yes ☐ No
3	BMI	☐ >23<br>☐ 18.5-23<br>☐ <18.5<br>☐ DK/refuse
4	Age	Record age<br>____________<br>☐ DK/refuse
5	Sex	☐ Male<br>☐ Female<br>☐ Not listed<br>☐ DK/refuse
6	Do you currently drive?	☐ Yes<br>☐ No<br>☐ DK/refuse
7	Do you live alone?	☐ No<br>☐ Yes<br>☐ DK/refuse
8	Dwelling	☐ House/apartment<br>☐ Retirement home/assisted living<br>☐ LTC/nursing home<br>☐ Temporary housing/other<br>☐ DK/refuse

(COGNITIVE SCREENING)<br>Administrator: *Do not cue participant. Instructions may be repeated once. "I would like to ask you some questions that ask you to use your memory"*		
9	Ottawa 3DY	Please check off which questions the participant answered correctly:<br>☐ What is the date?<br>☐ What day of the week is it?<br>☐ Spell the word WORLD backwards.<br>☐ What year is it?<br>☐ Could not attempt.
(SELF-REPORTED HEALTH)<br>Administrator: *Please enter the participant's response or have the participant/caregiver self-complete on device. If the participant did not pass the cognitive screening, please include the input of the caregiver.*		
10	How would you rate your general health?	☐ Excellent<br>☐ Fair<br>☐ Poor<br>☐ DK/refuse
11	Compared to one year ago, how would you rate your health now?	☐ The same or better<br>☐ Somewhat worse<br>☐ Much worse<br>☐ DK/refuse
12	Do you have pain that interferes with your usual activities or sleep?	☐ No<br>☐ Yes<br>☐ Sometimes<br>☐ DK/refuse
(NUTRITIONAL STATUS)		
13	Are you eating less than you normally would over the past year?	☐ No<br>☐ Yes, a little<br>☐ Yes, a lot<br>☐ DK/refuse

14	*If YES to previous question* → What is the reason you are eating less than you normally would?	☐ I have no appetite or I get full quickly<br>☐ I get indigestion or an upset stomach<br>☐ I have chewing or swallowing difficulty<br>☐ I have difficulty preparing meals<br>☐ I don't like the taste of food<br>☐ Other: ________________ ________________<br>☐ DK/refuse
15	In the past month, have you ever been hungry because you did not have enough available food?	☐ No<br>☐ Yes<br>☐ DK/refuse
16	Have you lost weight in the last year without trying?	☐ No<br>☐ Yes<br>☐ DK/refuse
17	5% Weight Loss	☐ No<br>☐ Yes<br>☐ DK/refuse
18	Do you eat protein at most meals? (e.g. chicken, meat, seafood, eggs, nuts, milk, cheese, beans, or protein supplements)	☐ No<br>☐ Yes<br>☐ DK/refuse
<u>(DAILY FUNCTION)</u>		
19	Do you have difficulty seeing (even when you are wearing glasses or contacts)?	☐ No<br>☐ Yes, a little<br>☐ Yes, a lot<br>☐ DK/refuse
20	Do you wear a hearing aid?	☐ No<br>☐ Yes<br>☐ DK/refuse
21	Do you have difficulty hearing in a conversation (even if you are wearing a hearing aid)?	☐ No<br>☐ Yes, a little<br>☐ Yes, a lot<br>☐ DK/refuse

22	Do you have trouble sleeping at night or staying asleep at night?	☐ No<br>☐ Yes, sometimes<br>☐ Yes, a lot<br>☐ DK/refuse
23	Do you ever have incontinence?	☐ Rarely or never<br>☐ Sometimes<br>☐ Often or always<br>☐ DK/refuse
24	Do you leak urine if you don't get to a toilet on time?	☐ Rarely or never<br>☐ Sometimes<br>☐ Often or always<br>☐ I have a catheter<br>☐ DK/refuse
25	Do you leak stool if you don't get to a toilet on time?	☐ Rarely or never<br>☐ Sometimes<br>☐ Often or always<br>☐ DK/refuse
**For each activity, indicate whether or not you can do it with or without help:**		
26	Bathing/showering	☐ Without help<br>☐ With help<br>☐ DK/refuse
27	Getting in and out of bed	☐ Without help<br>☐ With help<br>☐ DK/refuse
28	Getting dressed (not including socks)	☐ Without help<br>☐ With help<br>☐ DK/refuse
29	Going to the toilet	☐ Without help<br>☐ With help<br>☐ DK/refuse
30	Using telephone/cell phone	☐ Without help<br>☐ With help<br>☐ DK/refuse
31	Taking my medications (at the correct time and the correct dose)	☐ Without help<br>☐ With help<br>☐ DK/refuse
32	Preparing food	☐ Without help<br>☐ With help<br>☐ DK/refuse

33	Getting around in the community	☐ Without help<br>☐ With help<br>☐ DK/refuse
(SOCIAL RELATION & SUPPORT)		
34	Do you do things regularly with others?	☐ Yes frequently (daily or weekly)<br>☐ Sometimes (at least monthly)<br>☐ Almost never<br>☐ DK/refuse
35	Do you ever feel left out or alone?	☐ A lot<br>☐ Sometimes<br>☐ Almost never<br>☐ DK/refuse
36	Do you have enough people in your life you can ask for help when you need it?	☐ No<br>☐ Yes<br>☐ DK/refuse
(MOOD)		
37	Do you ever feel sad?	☐ Very little or never<br>☐ Sometimes<br>☐ A lot<br>☐ DK/refuse
(ENERGY & PHYSICAL ACTIVITY)		
38	Do you have enough energy to do the things you want to do?	☐ No<br>☐ Sometimes<br>☐ Yes<br>☐ DK/refuse
39	How often do you engage in activities that require a moderate or vigorous level of energy? *(Examples: brisk walking, yard/house work, shoveling, gardening, dancing)*	☐ Hardly ever or never<br>☐ One to three times a month<br>☐ Once a week<br>☐ Two or more times a week<br>☐ DK/refuse
40	Have you stopped doing activities that are important to you because of your health?	☐ No<br>☐ Somewhat stopped<br>☐ Yes, completely<br>☐ DK/refuse

(STRENGTH & MOBILITY)		
41	Do you use any of the following walking aids? (*Not scored*)	☐ Cane<br>☐ Walker<br>☐ Wheelchair<br>☐ None of the above<br>☐ DK/refuse
42	Are you able to walk around the block? (*A "block" is about 400m or ¼ mile, e.g. running track)*	☐ Not able<br>☐ Yes, with difficulty or help<br>☐ Yes, with ease<br>☐ DK/refuse
43	Have you fallen in the last six (6) months?	☐ Yes, twice or more<br>☐ Once<br>☐ None<br>☐ DK/refuse
44	Do you think you can stand on one foot?	☐ Yes, for 10 seconds or more<br>☐ Yes, for less than 10 seconds<br>☐ No, cannot do that<br>☐ DK/refuse
45	Can you climb a flight of stairs?	☐ Not able<br>☐ Yes, with difficulty or help<br>☐ Yes, with ease<br>☐ DK/refuse
46	Are you able to lift and carry items weighing 10lbs/4.5kg (*e.g. large bag of flour*)	☐ No, not able<br>☐ Yes, with difficulty or help<br>☐ Yes, with ease<br>☐ DK/refuse
(COGNITION)		
47	Do you have any difficulty remembering things or solving problems?	☐ Rarely<br>☐ Sometimes<br>☐ Almost always<br>☐ DK/refuse
Who provided the information for this section?		☐ Participant<br>☐ Caregiver<br>☐ Participant/ caregiver together

(MEDICAL HISTORY)<br>*Option 1: Ask the participant (if the participant did not pass cognitive screening, please include input of caregiver)*<br>*Option 2: Administrator completes based on health record.*<br>**Has a doctor ever diagnosed you with or treated you for any of the following?**		
48	Alzheimer's Disease or Dementia	☐ No<br>☐ Yes<br>☐ DK/refuse
49	Anxiety	☐ No<br>☐ Yes<br>☐ DK/refuse
50	Arthritis<br>*(Osteoarthritis – pain or stiffness in joints or Rheumatoid arthritis)*	☐ No<br>☐ Yes<br>☐ DK/refuse
51	Cancer *(any type, in past 5 years)*	☐ No<br>☐ Yes<br>☐ DK/refuse
52	Depression	☐ No<br>☐ Yes<br>☐ DK/refuse
53	Diabetes (type 1 or 2)	☐ No<br>☐ Yes<br>☐ DK/refuse
54	Gastrointestinal Disease *(Gastroesophageal Reflux Disease [GERD], Peptic Ulcer Disease [gastric or duodenal ulcer], Inflammatory Bowel Disease [Crohn's disease or ulcerative colitis])*	☐ No<br>☐ Yes<br>☐ DK/refuse
55	Heart Disease (*angina, myocardial infarction [heart attack], atrial fibrillation/other arrhythmia, or heart failure)*	☐ No<br>☐ Yes<br>☐ DK/refuse
56	Hypertension *(high blood pressure)*	☐ No<br>☐ Yes<br>☐ DK/refuse
57	Chronic Kidney Disease	☐ No<br>☐ Yes<br>☐ DK/refuse

58	Lung disease<br>*(Chronic Obstructive Pulmonary Disease [COPD], asthma, or Chronic Bronchitis)*	☐ No<br>☐ Yes<br>☐ DK/refuse
59	Osteoporosis or previous fracture (after age 40)	☐ No<br>☐ Yes<br>☐ DK/refuse
60	Parkinson's Disease or other Neurologic disease (*Multiple Sclerosis, epilepsy. Note: do NOT include Alzheimer's disease here*)	☐ No<br>☐ Yes<br>☐ DK/refuse
61	Stroke *(Cerebrovascular accident or transient ischemic attack)*	☐ No<br>☐ Yes<br>☐ DK/refuse
62	Thyroid Disease	☐ No<br>☐ Yes<br>☐ DK/refuse

TOTAL SCORE

Enter the final score here:	
Fit-Frailty Index Score<br>*(number between 0 and 0.7)* →	Fit-Frailty Score:

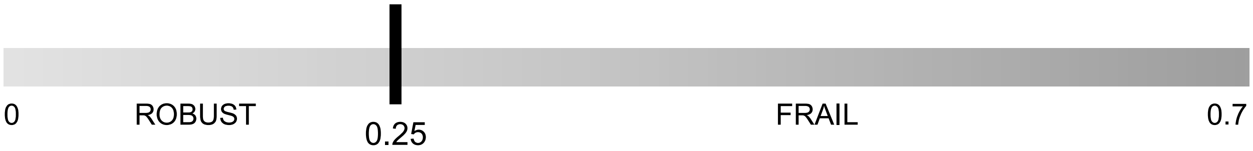

The resulting 'Fit-Frailty Score' is between 0 and 0.7. A score of 0 may represent full health and a score of 0.7 represents maximal frailty. A score of 1.0 is theoretically possible, however, >99% of people have scores below 0.7. In previous studies, scores above 0.25 are indicative of the "frail zone". Scores above 0.4 indicate severe frailty with an elevated chance of poor outcomes including mortality.

______________________	______________________	**Date**: ______/_______/_____
**CRF Completed By**	**Signature**	**YYYY MM DD**
